# Rapid screening of urinary tract infections: quantitative flow cytometry versus dipstick testing in a hospital cohort

**DOI:** 10.1007/s15010-026-02920-w

**Published:** 2026-08-11

**Authors:** B. Dirk, T. Hwandih, M. Holz, B. Schönbeck, L. von Müller

**Affiliations:** 1 Christophorus Kliniken GmbH, Südring 10, 48653 Coesfeld, Germany; 2 Faculty of Medicine, Saarland University, Homburg, Germany; 3 Sysmex Europe SE, Hamburg, Germany

**Keywords:** Urinary tract infection, Flow cytometry, Urine dipstick, Bacteriuria, Diagnostic screening, Diagnostic stewardship

## Abstract

**Purpose:**

We compared urine dipstick testing (DS) with quantitative flow cytometry (FC) and evaluated a diagnostic algorithm to optimise urinary tract infection (UTI) screening.

**Methods:**

In this single-centre study, 5,124 consecutive urine samples were analysed in parallel using DS and FC. Semi-quantitative urine culture was performed for samples with bacterial count (BC) ≥ 100/µL; samples below this threshold were classified as non-UTI without culture confirmation.

**Results:**

A BC ≥ 100/µL was observed in 2,281 samples (44.5%); among these, 1,368 were culture-confirmed as UTI, corresponding to 26.7% of all samples. FC outperformed DS, demonstrating higher sensitivity and specificity while reducing the need for follow-up cultures by 18%. The probability of UTI increased with rising BC and exceeded 90% at BC values ≥ 3,000/µL, particularly in samples without evidence of contamination. BC was significantly higher in Gram-negative than in Gram-positive infections (median 19,610 vs. 679/µL). Sample contamination was associated with elevated squamous cell counts (SQC ≥ 10/µL).

**Conclusion:**

FC with direct bacterial detection demonstrates superior performance compared with DS for both ruling in and ruling out UTIs. Quantitative FC parameters enable early estimation of UTI probability and sample contamination prior to culture results, supporting diagnostic stewardship and enabling earlier targeted clinical decision-making.

**Supplementary Information:**

The online version contains supplementary material available at https://doi.org/10.1007/s15010-026-02920-w.

## Introduction

UTIs are among the most common infectious diseases, affecting approximately 400 million individuals worldwide each year, with a substantial associated mortality burden [1]. The incidence has increased in various settings over recent decades, although the underlying reasons remain unclear [1, 2].

UTIs occur approximately six times more frequently in women than in men [1], and women have a lifetime risk of 50–60% [3]. This difference is largely attributable to anatomical differences, including a shorter urethra and the proximity of the vaginal and anal regions. A Lactobacillus-dominated microbiome, strongly influenced by hormonal status, is associated with a low vaginal pH and is considered a protective factor against infection by uropathogens [4–6].

Clinical presentation, diagnostic requirements, and treatment strategies differ substantially between outpatient and hospital settings. Uncomplicated lower UTIs (cystitis) in otherwise healthy, non-pregnant women are localised and may not require laboratory investigation [7]. Standard treatment includes increased fluid intake, analgesia and/or empirical oral antibiotic therapy. Despite treatment, 20–30% of women experience recurrence within 3–4 months [3], which is associated with reduced quality of life and adverse mental health outcomes [8, 9].

Microbiological diagnostics are not routinely required for uncomplicated UTIs, defined as infections in non-pregnant premenopausal women without anatomical or functional abnormalities. In contrast, UTIs in men are generally considered complicated due to possible prostate involvement, necessitating specific diagnostics including urine culture testing [7, 10].

In the hospital setting, UTIs are typically complicated and may be associated with systemic infection. Pyelonephritis and urosepsis represent severe forms with a substantial disease burden. Increased mortality has been associated with advanced age, comorbidities, and antimicrobial resistance [1, 2]. Therefore, early diagnosis using sensitive methods is essential for all complicated UTIs to enable prompt empirical treatment.

Bacterial culture has long been the diagnostic reference standard for UTI detection [10]. Culture enables semi-quantitative analysis of viable bacteria after overnight incubation. Prior antibiotic exposure may reduce culture sensitivity, whereas specificity may be impaired by contamination. Positive cultures are followed by identification using MALDI-TOF mass spectrometry and susceptibility testing, which requires additional incubation. Consequently, final results are typically available 2–3 days after sampling.

Diagnosis based solely on clinical symptoms has been associated with substantial diagnostic error rates [11]. Therefore, initial screening using point-of-care testing is essential to rapidly rule in or rule out UTIs. The most common screening method is the urine DS, which relies on semi-quantitative enzymatic reactions detecting indirect markers of UTI such as leucocyte esterase (LE) and nitrite. However, the reliability of these markers is limited and remains controversial, particularly in patients with limited leucocyte reactivity [12].

Screening for bacteriuria is also recommended in specific clinical scenarios, such as pregnancy and prior to urological procedures [13]. While DS may lack sufficient sensitivity to safely exclude UTI [7], routine culture of all samples is time-consuming, costly and delays diagnosis.

According to the EFLM European urinalysis guideline, DS is classified as a Level 1 (point-of-care) method, whereas particle counting is considered a Level 2 (routine laboratory) method [7]. FC is a modern technique that enables direct quantitative detection of bacteria and other urinary particles. Very low BC values measured by FC have been shown to be associated with a very low probability of UTI and may therefore reduce the need for confirmatory culture [14].

The aim of this study was to directly compare DS, FC and urine culture as the diagnostic reference standard, and to develop an optimised screening algorithm based on quantitative BC. This approach aims to enable early exclusion of non-infected patients while limiting unnecessary cultures and identifying patients with a high probability of UTI who may benefit from early intervention.

## Materials and methods

### Study design and urine samples

This single-centre observational study was conducted at a major regional hospital in Germany. Between May and October 2024, consecutive urine samples obtained from patients with suspected UTI were analysed as part of routine diagnostics. Samples were collected in 10-mL urine collection tubes (Sarstedt, Nümbrecht, Germany) following standard clinical urine collection procedures.

Semi-quantitative DS testing and quantitative FC were performed in parallel for all samples immediately upon arrival in the laboratory.

### Dipstick analysis

Semi-quantitative DS analysis was performed using the automated UC-3500 system (Sysmex, Kobe, Japan) with MEDITAPE UC-9 A strips. For the present analysis, parameters relevant for UTI screening were evaluated, including LE, nitrite, haemoglobin and protein. Other DS parameters not directly related to infection diagnostics were excluded from further analysis.

### Flow cytometry

Quantitative urine particle analysis was performed using the UF-5000 flow cytometer (Sysmex, Kobe, Japan). Native urine samples were analysed without prior sample preparation. Quantitative detection of urinary particles was completed within 1 min using laser-based fluorescence technology (488 nm) with multichannel detection.

Particles were classified according to size, granularity and fluorescence characteristics, enabling quantitative measurement of BC, leucocyte count (LC), erythrocyte count (EC), SQC, casts and crystals. The UF-5000 system additionally provides a Gram flag indicating Gram-related bacterial characteristics.

### UTI score

A multiparametric exploratory UTI score was calculated based on quantitative FC parameters, including BC, LC, SQC and Gram flag. The score was derived using predefined probability categories based on observed FC distributions and principles adapted from random forest-based variable weighting. Detailed score composition is provided in Supplementary File 1.

### Urine culture

Semi-quantitative urine culture was performed for all samples with BC ≥ 100/µL according to the established routine diagnostic algorithm. Samples below this threshold were not routinely cultured, as previous studies have shown a negligible number of clinically relevant UTIs at very low BC [15, 16].

Culture was performed using Columbia blood agar and MacConkey agar (Becton Dickinson, Heidelberg, Germany) with unprocessed and 1:100 diluted samples. Bacterial identification was performed using MALDI-TOF mass spectrometry (Bruker Daltonics, Bremen, Germany), and antimicrobial susceptibility testing was performed using Vitek 2 microdilution assay (bioMérieux, Marcy-l’Étoile, France).

Samples were classified as UTI, non-UTI or contamination according to culture criteria of national and European microbiological guidelines (MiQ and EFLM/ESCMID criteria) [7, 10].

### Statistical analysis

Data were analysed using GraphPad Prism version 6 and MedCalc^®^ Statistical Software version 22.021 (MedCalc Software Ltd, Ostend, Belgium). Continuous variables are presented as median and interquartile range (IQR), and categorical variables as proportions.

Comparisons between independent groups were performed using the chi-squared test. Continuous variables were compared using the Mann–Whitney U test. A two-sided p-value < 0.05 was considered statistically significant.

Diagnostic performance of DS and FC parameters was evaluated using culture-confirmed UTI as the reference standard. Sensitivity, specificity and follow-up culture rates were calculated for predefined thresholds. Receiver operating characteristic (ROC) analysis was conducted to assess discrimination of BC, LC, SQC and the combined UTI score.

## Results

### Population

A total of 5,124 consecutive routine urine samples from patients treated at a major regional hospital in Germany were included. The cohort comprised 58.2% women and 41.8% men (*p* < 0.001). The median age was 65.8 years (IQR: 35.7–81.5), with no significant difference between sexes (*p* = 0.18) (Table 1).

**Table 1:** Patient characteristics and classification of UTI

Sex	Samples (*n*)	Age, years Median (IQR)	UTI (*n*, %)	Non-UTI (*n*, %)	Contamination (*n*, %)
All	5,124	65.8 (35.7–81.5)	1,368 (26.7% RT)	2,918 (56.9% RT)	838 (16.4% RT)
Men	2,059	67.6 (43.3–80.4)	328 (15.9% RT; 24% CT)	1,651 (80.2% RT; 56.5% CT)	80 (3.9% RT; 9.5% CT)
Women	3,065	63.2 (33.8–82.4)	1,040 (33.9% RT; 76% CT)	1,267 (41.3% RT; 43.4% CT)	758 (24.7% RT; 90.5% CT)
p-value	< 0.001	0.180	< 0.001	< 0.001	< 0.001

RT = row total; CT = column total. p-values compare men versus women

### Direct comparison of FC and DS with culture

A total of 2,843 samples (55.5%) had BC values below the threshold (< 100/µL) and were classified as non-UTI without further culture analysis. The remaining 2,281 samples (44.5%) had elevated BC and underwent confirmatory culture according to national and international guidelines (MiQ, EFLM/ESCMID).

Of these, 1,368 samples (60.0%) were classified as UTI and 838 (36.7%) as contaminated. Seventy-five samples (3.3%) remained culture-negative (non-UTI) despite BC ≥ 100/µL as measured by FC.

Overall, 2,918 samples (56.9%) were classified as non-UTI (including BC < 100/µL and culture-negative samples with BC ≥ 100/µL), 1,368 (26.7%) as confirmed UTIs and 838 (16.4%) as contaminated. Most confirmed UTIs were monomicrobial, caused by Gram-negative uropathogens (*n* = 1,071, 78.3%). In addition, infections involving two uropathogens (*n* = 190, 13.9%) and monomicrobial Gram-positive infections (*n* = 107, 7.8%) were observed.

Contaminated samples were defined as those with ≥ 3 species (*n* = 189, 22.6%), ≤ 10³ CFU/mL (*n* = 79, 9.4%), skin bacteria (*n* = 566, 67.5%) or yeast (*n* = 4, 0.5%). The frequency of UTIs and of contamination was higher in women than in men (*p* < 0.001) (Table 1).

### Direct comparison of DS and FC

The predictive performance of DS and FC for culture-confirmed UTI was compared (Table 2). Both methods were performed within the same diagnostic workflow (UC-3500, UF-5000), and both results were available within minutes.

**Table 2:** Performance of initial screening tests for UTI (*n* = 5,124) using dipstick (DS) and flow cytometry (FC)

Urine screening test	Sensitivity (%)	Specificity (%)	Follow-up cultures (*n* (%))
**Semi-quantitative dipstick (DS; UC-3500)**			
Nitrite	39.8	99.3	571 (11.1%)
Leucocyte esterase ≥+ (LE low)	88.5	58.3	2,775 (54.2%)
Leucocyte esterase ≥++ (LE high)	76.4	77.4	1,895 (37.0%)
Combined DS results			
Nitrite and/or leucocyte esterase (LE low)	90.3	58.2	2,804 (54.7%)
Nitrite and/or leucocyte esterase (LE high)	82.3	77.2	1,983 (38.7%)
**Quantitative flow cytometry (FC; UF-5000)**			
Bacterial count ≥ 100/µL (BC low)	100.0	75.7	2,281 (44.5%)
Bacterial count ≥ 300/µL (BC elevated)	91.2	86.1	1,769 (34.5%)
Bacterial count ≥ 1,000/µL (BC high)	79.2	93.7	1,321 (25.8%)
Bacterial count ≥ 10,000/µL (BC very high)	55.0	99.0	791 (15.4%)
Leucocyte count ≥ 10/µL (LC low)	92.5	52.5	3,049 (59.5%)
Leucocyte count ≥ 40/µL (LC elevated)	77.6	78.2	1,880 (36.7%)
Leucocyte count ≥ 100/µL (LC high)	63.6	88.2	1,314 (25.6%)

According to the study design, urine cultures were performed only in samples with BC ≥ 100/µL. Therefore, culture-confirmed UTIs were by definition restricted to these samples. As a result, the sensitivity of FC using this threshold was 100% within the constraints of the study design.

A lower sensitivity was observed for FC using LC (≥ 10/µL; 92.5%) and for DS using LE (88.5%). Nitrite testing (DS) resulted in the lowest sensitivity (39.8%) but with very high specificity (99.3%). The combination of LE and nitrite only marginally increased sensitivity (88.5% vs. 90.3%).

Notably, the FC screening approach with the highest sensitivity (BC ≥ 100/µL) required fewer follow-up cultures than DS using LE (44.5% vs. 54.2%).

FC enables quantitative analysis of urinary particles, whereas DS provides only semi-quantitative results. Increasing the DS threshold level (LE ≥++) resulted in reduced sensitivity (76.4%), falling below the 80% threshold generally considered desirable for a screening test.

Both BC and LC correlated with the probability of UTI, which exceeded 90% at BC values ≥ 3,000/µL. Sensitivity and specificity cut-offs for various BC and FC values are provided in Supplementary File 2.

ROC curve analysis demonstrated that BC (AUC = 0.846) discriminated UTIs better than LC (AUC = 0.714). SQC was a negative predictor of UTI (AUC = 0.721) and a positive predictor of contamination. High SQC (≥ 10/µL) strongly indicated contamination, particularly when BC was only moderately elevated (< 3,000/µL) (Table 3). EC showed no association with UTI.

**Table 3:** Association of contamination and UTI rates with SQC and BC

	*n*	Contamination	Non-UTI	UTI
Squamous cell count < 10/µL (SQC low)				
Bacterial count (BC): 100–399/µL	245	51.8%	9.0%	39.2%
Bacterial count (BC): 400–999/µL	95	29.5%	5.3%	65.3%
Bacterial count (BC): 1,000–2,999/µL	88	14.8%	2.3%	83.0%
Bacterial count (BC): ≥3,000/µL	602	3.2%	0.7%	96.2%
Squamous cell count ≥ 10/µL (SQC high)				
Bacterial count (BC): 100–399/µL	383	76.0%	6.5%	17.5%
Bacterial count (BC): 400–999/µL	237	70.9%	3.8%	25.3%
Bacterial count (BC): 1,000–2,999/µL	193	66.8%	2.1%	31.1%
Bacterial count (BC): ≥3,000/µL	438	14.4%	0.9%	84.7%

High SQC is associated with increased contamination rates, except at very high BC values (≥ 3,000/µL). In samples with low SQC (< 10/µL), low BC values are still associated with a considerable proportion of contamination, whereas higher BC values predominantly indicate true UTIs

Overall, the lower sensitivity of DS resulted in underdiagnosis of culture-confirmed UTIs compared with FC. Samples missed by DS frequently demonstrated high bacterial growth in culture (more than 58% showed CFU ≥ 10⁶/mL), which supports their classification as false negatives.

### Comprehensive analysis of FC: UTI score versus BC

**Fig. 1:**
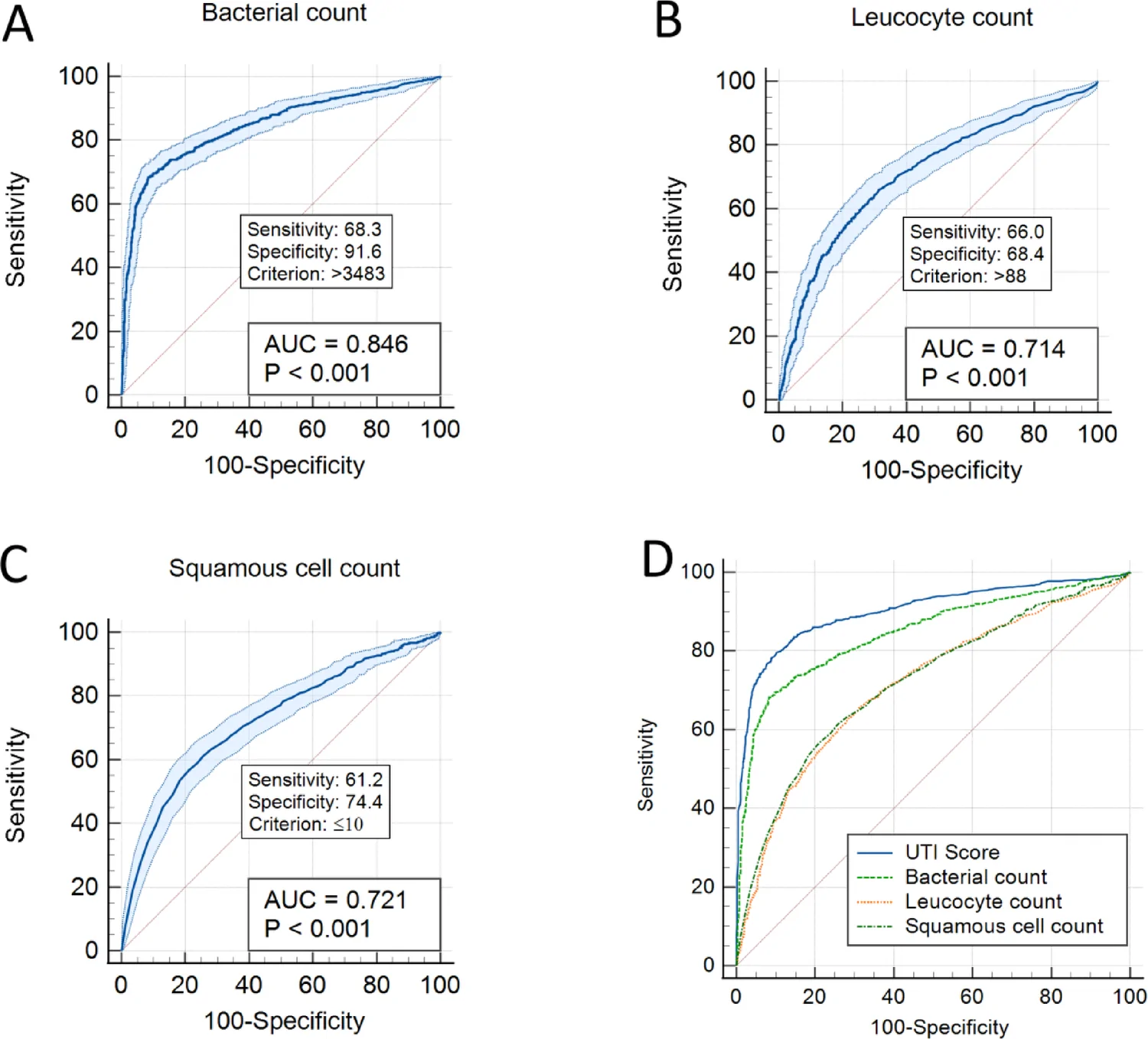
Performance of flow cytometry parameters at different thresholds. ROC curve analysis was conducted for: **A** bacterial count (BC), **B** leucocyte count (LC), **C** squamous cell count (SQC), and **D** combined parameters including the UTI score

A multiparametric exploratory UTI score (range 0–100) based on quantitative FC parameters demonstrated excellent discrimination of UTIs (AUC = 0.901) compared with individual parameters (Fig. 1). However, its sensitivity was lower than that of BC ≥ 100/µL alone (93.9% vs. 100%). UTI score values were associated with the likelihood of culture-confirmed infection. For example, a low UTI score < 15 was very unlikely to represent UTI (probability < 15%) and a UTI score > 55 indicated a high likelihood of UTI (specificity > 85%). Gradual differences can be derived from ROC curve analysis (Supplementary File 2).

### Uropathogens

Monomicrobial infections (> 10³ CFU/mL) were predominantly caused by *Escherichia coli*, followed by *Klebsiella* spp., *Proteus* spp., *Pseudomonas aeruginosa* and other Gram-negative bacteria (Table 4). Gram-positive infections were less frequent and mainly involved *Enterococcus* spp.

**Table 4:** Sex-specific distribution of uropathogens in UTIs

	All		Men		Women		
	*n*	%	*n*	%	*n*	%	*p*-value
Uropathogens (total)	**1178**	**100%**	**269**	**100%**	**909**	**100%**	NA
*Escherichia coli*	741	62.9%	97	36.1%	644	**70.8%**	< 0.0001
*Klebsiella spp.*	132	11.2%	36	13.4%	96	10.6%	ns
*Proteus spp.*	86	7.3%	40	**14.9%**	46	5.1%	< 0.0001
*P. aeruginosa*	35	3.0%	22	**8.2%**	13	1.4%	< 0.0001
*Citrobacter spp.*	32	2.7%	14	**5.2%**	18	2.0%	0.0087
*Enterobacter spp.*	23	2.0%	9	3.3%	14	1.5%	ns
*Serratia spp.*	9	0.8%	8	**3.0%**	1	0.1%	< 0.0001
*Morganella spp.*	4	0.3%	4	**1.5%**	0	0.0%	0.0027
Other Gram-negative bacteria	9	0.8%	4	1.5%	5	0.6%	ns
*Enterococcus spp.*	65	5.5%	25	**9.3%**	40	4.4%	0.0035
*S. agalactiae (GBS)*	15	1.3%	1	0.4%	14	1.5%	ns
*A. urinae/sanguinicola*	12	1.0%	6	**2.2%**	6	0.7%	0.0354
*S. saprophyticus*	4	0.3%	0	0.0%	4	0.4%	ns
Other Gram-positive bacteria	11	0.9%	3	1.1%	8	0.9%	ns

Differences were analysed using Fisher’s exact test; p-values are shown

Further analysis of FC parameters demonstrated that BC was significantly higher in Gram-negative (median 19,610/µL, IQR 3,098–43,568) than in Gram-positive infections (median 679/µL, IQR 343–2,381) and contaminated samples (median 400/µL, IQR 181–1,078).

**Fig. 2:**
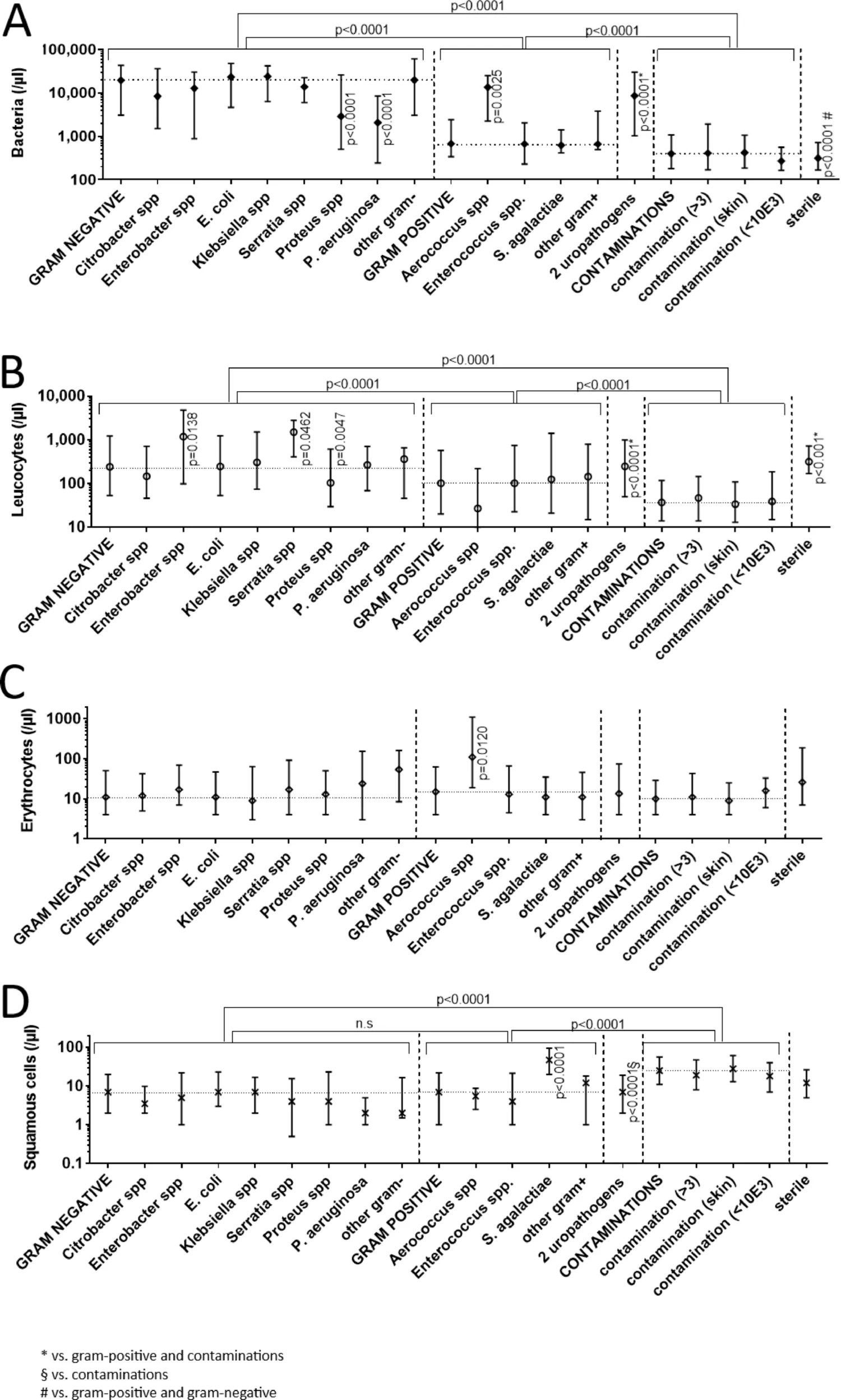
Quantitative characterisation of cell types in urine by flow-cytometry. Groups were defined by culture results. Differences between major groups (Gram-negative, Gram-positive, mixed infections, contamination) but also single species are shown. Statistical analysis (Mann-Whitney U test) was conducted for major groups. Bacterial count (BC) (**A**), leucocyte count (LC) (**B**), erythrocyte count (EC) (**C**), and squamous cell count (SQC) (**D**) are shown

In infections involving two uropathogens, BC (median 8,663/µL, IQR 1,052–29,732) was similar to Gram-negative infections. For *Proteus* spp. and *P. aeruginosa*, BC was lower than typical Gram-negative infections. For infections with *Aerococcus* spp., BC was higher than other Gram-positive organisms (Fig. 2A).

LC, reflecting urinary inflammation, was higher in Gram-negative infections than in Gram-positive, contaminated or culture-negative samples (Fig. 2B). LC values in samples with two uropathogens resembled those of Gram-negative infections. Elevated LC was particularly observed in infections with *Enterobacter* and *Serratia* spp. LC was also elevated in culture-negative samples, possibly as a sign of recent UTI with prior antibiotic treatment; however, this hypothesis requires further investigation.

EC did not differ significantly between groups (Fig. 2C), except for *Aerococcus* infections with elevated EC.

SQC did not differ between Gram-negative, Gram-positive and mixed infections (2 uropathogens) but was significantly higher in contaminated samples (Fig. 2D). Elevated SQC was also observed in samples with group B streptococci (*S. agalactiae; GBS*), suggesting that most GBS cases may represent contamination rather than true infection.

### Sex and age differences

The prevalence of UTIs (33.9% vs. 15.9%) and contamination (24.7% vs. 3.9%) was higher in women than in men (*p* < 0.001) (Table 1). UTIs in women were predominantly caused by *E. coli*, whereas infections in men demonstrated a more heterogeneous pathogen distribution (Table 4).

In men, there was a higher proportion of healthcare-associated Gram-negative bacteria with increased antimicrobial resistance (KES group: e.g. *Klebsiella*, *Enterobacter*, *Serratia*), *P. aeruginosa*, and Gram-positive bacteria (e.g. *Enterococcus*, *Aerococcus*).

**Fig. 3:**
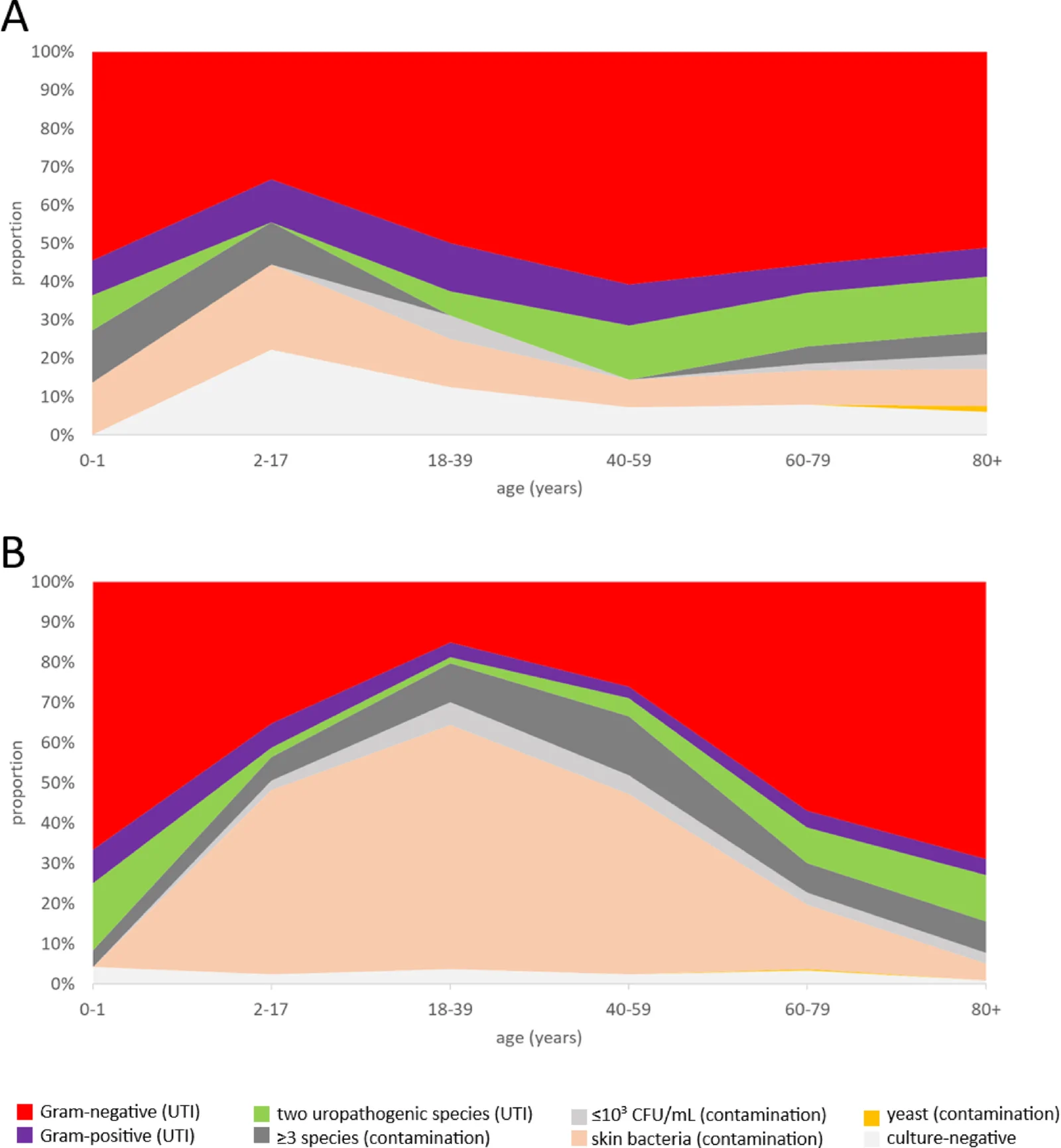
Distribution of UTI, contamination and culture-negative samples among cases with BC ≥ 100/µL. Differences between men (**A**) and women (**B**) across age groups are shown Contamination rates were significantly higher in women than in men, with marked age-related differences. Notably, the highest contamination rates were observed in younger women (18–39 years), rather than in older women or infants

Unexpectedly, contamination in women (Fig. 3) was more common in younger and middle-aged groups rather than in older age groups with increased frailty. In men, contamination rates remained low and relatively constant across various age groups.

## Discussion

The aim of this study was to optimise the diagnostic screening algorithm for UTIs, enabling rapid, highly sensitive detection within minutes, while minimising the need for confirmatory cultures. High sensitivity is a fundamental requirement for an effective screening test [7, 17]. However, there is no universally accepted threshold for diagnostic sensitivity, as it depends on factors such as screening algorithm (unselected vs. selected populations), test availability, disease prevalence and the clinical consequences of underdiagnosis. In practice, a sensitivity of 90% is generally considered to be acceptable for diagnostic tests in symptomatic infections. Screening tests are typically followed by confirmatory assays with high specificity.

In the present study, we compared conventional DS with modern FC for rapid UTI screening, using culture as the diagnostic reference standard [10]. In patients with severe UTI, the impact of culture results on treatment initiation is often limited due to the prolonged turnaround time (48–72 h). Although experimental approaches such as direct disc diffusion testing [18] or rapid liquid culture [19] may shorten turnaround time, they still require incubation and conventional culture confirmation. Furthermore, contamination due to improper sampling is typically identified only after a delay of 2–3 days, limiting the clinical utility of culture alone.

Rapid diagnostic methods are therefore needed to distinguish between negative and potentially positive urine samples early. The results of initial screening will directly influence clinical decision-making. Sensitive screening strategies allow confirmatory cultures to be restricted to suspected samples and may guide decisions regarding early empirical antibiotic therapy. Both underdiagnosis (low sensitivity) and overdiagnosis (low specificity) should be minimised to reduce the risks of undertreatment and overtreatment [20].

For many years, urine DS testing based on LE and nitrite has been widely used despite its well-known limitations in sensitivity and specificity [17]. In contrast, newer methods enabling quantitative bacterial detection have become available and offer improved diagnostic performance [21].

In this study, we confirmed that surrogate markers of infection detected by DS (LE and nitrite) have limited diagnostic value due to suboptimal sensitivity and specificity, which was consistent with previous reports [16, 17]. A sensitivity of 90% was achieved only when LE and nitrite were combined. No single DS parameter met the threshold criteria for sensitivity expected for a reliable screening test. Moreover, reliance on leucocyturia is inherently problematic for the detection of UTI, as it depends on host immune response, which may be impaired in patients with leucocytopenia or immunosuppression.

Current international guidelines support the use of direct quantitative bacterial detection as a first-line approach. Indirect markers such as leucocytes are considered a secondary choice [7]. FC enables rapid quantitative assessment of bacteria and other urinary particles (LC, EC, SQC, crystals) within minutes and has recently been complemented by more advanced automated imaging systems [22]. In previous studies, FC appeared to be superior to imaging methods for quantitative bacterial detection with lower false-positive rates, whereas imaging may be advantageous for crystal characterisation and more detailed discrimination of morphological changes [23].

Our findings demonstrate the practical benefits of an optimised screening approach for both early rule-out and rule-in of UTIs. Within the constraints of the study design, a BC < 100/µL (FC) was associated with a very low probability of UTI and may allow a substantial proportion of samples (approximately 55%) to be excluded at an early stage from further testing. Compared with DS, this approach reduced the number of unnecessary cultures while maintaining higher sensitivity. Previous studies have proposed similar cut-off values of a BC ≥ 100/µL [13, 14, 19], which is consistent with our results. The optimised FC-based algorithm reduced unnecessary cultures by approximately 18% compared with combined DS criteria (surrogate markers).

Quantitative FC parameters were strongly associated with the probability of UTI. Higher BC values correlated with an increased likelihood of infection, which may facilitate early clinical decision-making, particularly with regard to empirical antibiotic therapy. We therefore suggest that quantitative BC values, together with an estimated probability of UTI, should be reported to clinicians to facilitate individualised disease management.

The combination of low BC (100–1,000/µL) with high SQC (≥ 10/µL) was a robust predictor of contamination (74%). As these parameters are available within minutes, early identification of samples with presumed contamination enables prompt repeat sampling of properly collected midstream urine samples, thus reducing diagnostic delay.

This study also demonstrated distinct quantitative profiles (FC) for Gram-negative and Gram-positive infections. These differences may reflect variations in bacterial growth (BC) and host cell response (LC). Growth kinetics and inflammation are influenced by pathogen-specific virulence factors and host characteristics [24]. Notably, infections involving two uropathogens showed similar FC profiles to monomicrobial Gram-negative infections, suggesting that such cases may represent true infections rather than contamination.

We also identified species-specific differences in FC parameters, including lower BC in infections caused by *Proteus* spp. and *P. aeruginosa*, and higher BC in *Aerococcus* spp. infections. Increased inflammatory response (LC) was particularly evident in infections with *Enterobacter* and *Serratia* spp. The underlying mechanisms for these differences remain unclear and warrant further investigation.

Interestingly, elevated SQC values were observed in samples with group B streptococci (*S. agalactiae*), closely resembling contamination patterns. This suggests that many such findings may reflect contamination rather than true infection. The clinical relevance of enterococci and GBS in UTIs is also discussed in other studies [25].

Significant sex-related differences were observed in infection rates, contamination rates and pathogen distribution. Women had higher rates of both UTIs and contamination, with *E. coli* predominating. In men, infections were more heterogeneous and included a higher proportion of more antimicrobial-resistant organisms, such as *P. aeruginosa*, KES group species and Gram-positive bacteria.

Unexpectedly, contamination rates were highest in younger and middle-aged women rather than in older individuals. This finding contrasts with expectations based on frailty and impaired sample collection in older patients. One possible explanation is a higher mucosal bacterial load in premenopausal women, although this hypothesis requires further confirmation. These findings highlight the importance of proper patient instruction for midstream urine collection, particularly in younger women.

Contamination leads to diagnostic uncertainty, delays and the need for repeat testing. Based on elevated SQC values, repeat sampling should be performed in cases with suspected contamination to improve diagnostic accuracy [26].

The exploratory UTI score demonstrated excellent discrimination but did not improve sensitivity compared with BC alone. In this study, direct bacterial detection with a low cut-off value remains the best unselected primary screening marker to rule out UTI. Multiparametric approaches are more complex and may contribute to secondary risk stratification. Recent studies have demonstrated the benefits of structured diagnostic algorithms to improve antimicrobial stewardship and clinical decision-making [21, 27]. Our results support the implementation of a stepwise diagnostic approach: first, exclusion of UTI in samples with BC < 100/µL; second, culture confirmation for samples above this threshold; and third, risk stratification of suspected samples based on quantitative FC values for bacteria and different host cells.

An excellent prediction of UTI by quantitative BC values was achieved for samples without squamous cell contamination (SQC < 10/µL) (Table 3). Samples with SQC ≥ 10/µL and with low, moderate and high BC (BC up to 3,000/µL) are at increased risk of contamination. Only very high BC values (> 3,000/µL) are highly predictive of UTI despite increased SQC (≥ 10/µL). A similar stratification of suspected samples can also be achieved using the UTI score, a multiparametric model based on quantitative FC parameters including BC and SQC (Supplementary File 1 and 2).

This risk-based approach may guide clinical decision-making, including the initiation of empirical antibiotic therapy in patients with a high probability of UTI, while encouraging a more cautious approach in low- and intermediate-risk cases, particularly when contamination is suspected.

This study has several important limitations. First, urine cultures were performed only in samples with BC ≥ 100/µL according to the routine diagnostic algorithm. Consequently, the study is subject to partial verification bias, as samples below this threshold were not microbiologically verified. This design may have resulted in an overestimation of the diagnostic performance of FC, particularly the apparent sensitivity at the predefined BC screening threshold. Therefore, the reported sensitivity should be interpreted within the context of the study design rather than as an absolute estimate. Prospective studies performing urine culture irrespective of FC findings are needed to determine the true diagnostic accuracy of this approach. Second, this was a single-centre observational study, which may limit generalisability to outpatient populations or other healthcare settings. Third, detailed clinical information – including symptoms, prior antibiotic therapy, catheterisation status, and other patient characteristics – was not systematically available. Finally, although the proposed UTI score showed promising discriminatory performance, it remains exploratory and requires independent external validation before routine clinical implementation.

The proposed diagnostic algorithm was designed for a hospital setting with a focus on high sensitivity to avoid underdiagnosis of infections. In outpatient settings predominantly characterised by *E. coli* infections in otherwise healthy individuals, a higher cut-off value (e.g. BC ≥ 400/µL) may be acceptable for ruling out uncomplicated UTIs. However, in hospitalised patients with complicated infections, immunosuppression and rare uropathogens, a higher threshold may compromise sensitivity, which may be particularly detrimental in Gram-positive infections with lower BC.

Further validation of this risk stratification approach is required in prospective, multicentre studies involving well-defined patient populations.

## Conclusion

Initial screening of urine samples using FC was superior to conventional DS due to its ability to directly detect and quantify bacteria, the primary cause of UTIs. Quantitative BC measurements may support early rule-out and rule-in strategies within minutes. However, the diagnostic performance of the proposed thresholds requires prospective validation using culture irrespective of FC findings.

The probability of a UTI correlates strongly with BC and, to a lesser extent, with LC. Risk stratification may become relevant for future therapeutic algorithms. In addition, early identification of contaminated samples through elevated SQC enables prompt repeat sampling of properly obtained urine samples, thus reducing diagnostic delay.

## Supplementary Information

Below is the link to the electronic supplementary material.

Supplementary Material 1

Supplementary Material 2

## Data Availability

All data supporting the findings of this study are available within the paper and its Supplementary Information. Additional data that support the findings of this study are available from the corresponding author upon reasonable request.
