# Supplementary material for: Rapid screening of urinary tract infections: quantitative flow cytometry versus dipstick testing in a hospital cohort
Source: Infection. 2026 Aug 11;54(5):2749–59. doi: 10.1007/s15010-026-02920-w (PMC13727914; doi:10.1007/s15010-026-02920-w)
Supplement: Supplementary file 1 [file 15010_2026_2920_MOESM1_ESM.docx]

**Supplementary File 1**
**Urinary Tract Infection (UTI) Score Development**

The development of the UTI score was inspired by principles borrowed from the random forest approach. However, three deliberate modifications were made in order to prioritise biological explainability. These modifications are explained below.

**1. Decision Tree Design**
Unlike conventional computer-generated random forest algorithms, which are typically composed of many small random decision trees, the UTI score algorithm is built on a foundation of two large structured decision trees (average depth of 5). Each tree was carefully designed to capture a defined biological domain relevant to UTI diagnostics. Each decision tree independently generates a likelihood estimate, and the final score is calculated as a weighted average of the two trees.

**2. Parameter Selection**
Each included parameter had a clear pathophysiological justification. This contrasts with traditional machine learning approaches, which often include parameters based primarily on statistical metrics. High statistical performance in the training dataset does not necessarily imply that a parameter is biologically appropriate for use in the decision tree.

**3. Parameter Cut-off Determination**
One of the pitfalls of purely data-driven algorithms is cut-off bias. The ‘best’ cut-off identified in the training dataset (based on area under the curve) is not always optimal. This is mainly because cut-offs are often influenced by demographic, biological, and epidemiological variables. To avoid this, UTI score algorithm cut-offs were defined and validated based on clinical applicability and published reference intervals from across the globe.

These three adjustments ensure that the algorithm captures the necessary biological variability while avoiding the overfitting commonly seen in purely computer-generated, data-driven models.

**Description of the Decision Trees**

**Decision Tree 1: Primary Diagnostic Classification**
Decision Tree 1 establishes the diagnostic foundation. Its core structure is anchored on four decision tree levels. The root node, or level 1 parameter, is the leucocyte count, which is key to identifying potential infection-driven inflammation. Leucocyte count is categorised into three groups (<40, 40–120, and >120 cells/µL).

The level 2 parameter is the squamous epithelial cell count, categorised into four groups (<1, 2–50, 50–100, and >100 cells/µL). Practically, the interaction of level 1 and level 2 parameters creates a 3 × 4 matrix with three main clusters. Samples classified as proven UTIs typically have high leucocyte counts (>120) and low squamous cell counts (<50), whereas contaminated samples have low leucocyte counts (<40) and high squamous cell counts (>50). Low counts in both parameters were strongly associated with UTI-negative samples.

The level 3 parameter (Gram stain flag) was introduced to improve specificity. It provides four main classifications: absence of a flag, Gram-negative flag, Gram-positive flag, and mixed Gram flag. The Gram-negative flag is highly specific for UTI, whereas the Gram-positive flag is strongly associated with contaminated samples.

The bacterial count constitutes level 4 and serves as a surrogate indicator of infection severity. It is categorised into low counts (<100), moderate counts (100–2000), and high counts (>2000). High counts are usually a hallmark of proven infection.

In most cases, these four levels provide clear separation, but some grey-zone nodes are further resolved using additional end-node parameters. At minimum, however, Decision Tree 1 forms a 3 × 4 × 4 × 3 matrix, yielding at least 144 final end nodes. Each end node is assigned a specificity-derived likelihood for predicting a UTI.

**Decision Tree 2** uses a similar four-level design. However, it is tailored to discriminate difficult-to-differentiate proven UTIs from contamination by introducing engineered features. These engineered parameters exploit opposing biological trends.

A simple example of such a parameter is the bacterial count-to-squamous cell ratio. Proven UTIs tend to have high bacterial counts and low squamous cell counts, whereas contaminated samples show lower bacterial counts with higher squamous cell counts. The ratio therefore demonstrates improved discriminatory performance in grey-zone cases compared with either parameter alone.

The final UTI score is calculated as the weighted average of Tree 1 and Tree 2.
