# Supplementary material for: Rapid screening of urinary tract infections: quantitative flow cytometry versus dipstick testing in a hospital cohort
Source: Infection. 2026 Aug 11;54(5):2749–59. doi: 10.1007/s15010-026-02920-w (PMC13727914; doi:10.1007/s15010-026-02920-w)
Supplement: Supplementary file 2 [file 15010_2026_2920_MOESM2_ESM.docx]

**Supplementary File 2**

Sensitivity and specificity cut-off values for ROC curve analysis. Only cases with bacterial count ≥100/µl were included.

| **Bacterial count (BC)** | | |
| --- | --- | --- |
| Criterion | Sensitivity | Specificity |
| ≥101 | 100 | 0 |
| >102 | 99.85 | 0.44 |
| >103 | 99.71 | 1.32 |
| >104 | 99.56 | 1.65 |
| >105 | 99.41 | 2.2 |
| >106 | 99.19 | 2.53 |
| >108 | 99.12 | 3.08 |
| >110 | 98.97 | 4.51 |
| >112 | 98.9 | 5.28 |
| >114 | 98.75 | 5.5 |
| >116 | 98.68 | 6.71 |
| >117 | 98.6 | 6.82 |
| >118 | 98.45 | 7.26 |
| >120 | 98.23 | 8.36 |
| >125 | 98.09 | 9.68 |
| >126 | 97.94 | 10.01 |
| >127 | 97.79 | 10.56 |
| >130 | 97.72 | 11.33 |
| >131 | 97.57 | 11.88 |
| >132 | 97.42 | 12.21 |
| >133 | 97.35 | 12.65 |
| >135 | 97.13 | 12.98 |
| >137 | 97.06 | 13.09 |
| >138 | 96.91 | 13.2 |
| >140 | 96.69 | 13.75 |
| >141 | 96.62 | 14.19 |
| >142 | 96.54 | 14.52 |
| >148 | 96.39 | 16.06 |
| >149 | 96.32 | 16.28 |
| >152 | 96.17 | 16.83 |
| >154 | 96.1 | 17.82 |
| >156 | 96.03 | 18.81 |
| >158 | 95.95 | 19.14 |
| >159 | 95.73 | 19.25 |
| >160 | 95.58 | 19.8 |
| >162 | 95.51 | 20.57 |
| >163 | 95.36 | 21.01 |
| >166 | 95.22 | 21.67 |
| >169 | 95.14 | 22.99 |
| >170 | 95.07 | 23.32 |
| >174 | 95 | 24.31 |
| >177 | 94.92 | 24.64 |
| >180 | 94.7 | 24.97 |
| >181 | 94.55 | 25.3 |
| >183 | 94.41 | 25.74 |
| >184 | 94.33 | 25.96 |
| >187 | 94.26 | 26.62 |
| >189 | 94.19 | 26.95 |
| >192 | 94.11 | 27.28 |
| >194 | 94.04 | 27.72 |
| >196 | 93.97 | 27.83 |
| >201 | 93.89 | 29.04 |
| >202 | 93.82 | 29.26 |
| >207 | 93.75 | 29.92 |
| >211 | 93.67 | 30.69 |
| >212 | 93.6 | 30.8 |
| >214 | 93.52 | 31.13 |
| >216 | 93.45 | 31.57 |
| >217 | 93.38 | 31.79 |
| >219 | 93.3 | 32.34 |
| >223 | 93.23 | 32.78 |
| >224 | 93.16 | 32.89 |
| >230 | 93.08 | 33.99 |
| >231 | 93.01 | 34.1 |
| >233 | 92.94 | 34.43 |
| >235 | 92.79 | 34.54 |
| >236 | 92.64 | 34.76 |
| >237 | 92.57 | 34.87 |
| >240 | 92.49 | 35.09 |
| >242 | 92.35 | 35.53 |
| >245 | 92.27 | 35.97 |
| >249 | 92.2 | 36.52 |
| >253 | 92.13 | 36.96 |
| >256 | 92.05 | 37.07 |
| >257 | 91.98 | 37.29 |
| >263 | 91.91 | 38.5 |
| >270 | 91.83 | 39.16 |
| >271 | 91.76 | 39.27 |
| >273 | 91.54 | 39.6 |
| >277 | 91.46 | 40.37 |
| >279 | 91.39 | 40.7 |
| >285 | 91.32 | 40.92 |
| >288 | 91.24 | 41.36 |
| >293 | 91.17 | 41.58 |
| >306 | 91.1 | 42.9 |
| >307 | 91.02 | 43.01 |
| >312 | 90.95 | 43.67 |
| >317 | 90.88 | 44.33 |
| >320 | 90.8 | 44.66 |
| >321 | 90.73 | 44.88 |
| >326 | 90.51 | 45.65 |
| >328 | 90.43 | 45.87 |
| >329 | 90.36 | 45.98 |
| >338 | 90.29 | 46.86 |
| >342 | 90.21 | 47.19 |
| >348 | 90.14 | 47.52 |
| >350 | 89.92 | 47.96 |
| >355 | 89.4 | 48.07 |
| >364 | 89.33 | 48.84 |
| >365 | 89.18 | 48.95 |
| >368 | 89.11 | 49.17 |
| >370 | 88.96 | 49.28 |
| >375 | 88.89 | 49.5 |
| >379 | 88.74 | 49.83 |
| >380 | 88.67 | 49.94 |
| >386 | 88.45 | 50.17 |
| >391 | 88.3 | 50.39 |
| >394 | 88.15 | 50.5 |
| >398 | 88.08 | 50.72 |
| >418 | 88.01 | 52.04 |
| >425 | 87.93 | 52.7 |
| >427 | 87.86 | 52.92 |
| >432 | 87.64 | 53.14 |
| >442 | 87.27 | 53.25 |
| >446 | 87.12 | 53.58 |
| >449 | 86.98 | 53.91 |
| >461 | 86.9 | 54.46 |
| >477 | 86.83 | 55.56 |
| >480 | 86.75 | 55.89 |
| >483 | 86.68 | 56 |
| >490 | 86.61 | 56.22 |
| >492 | 86.53 | 56.33 |
| >495 | 86.46 | 56.44 |
| >496 | 86.39 | 56.55 |
| >497 | 86.31 | 56.66 |
| >501 | 86.09 | 56.77 |
| >503 | 86.02 | 56.88 |
| >504 | 85.95 | 56.99 |
| >506 | 85.87 | 57.1 |
| >509 | 85.8 | 57.21 |
| >511 | 85.72 | 57.32 |
| >516 | 85.5 | 57.98 |
| >523 | 85.36 | 58.53 |
| >530 | 85.28 | 58.86 |
| >533 | 85.21 | 58.97 |
| >539 | 85.14 | 59.3 |
| >547 | 84.92 | 59.96 |
| >549 | 84.84 | 60.07 |
| >564 | 84.69 | 60.4 |
| >572 | 84.62 | 61.06 |
| >580 | 84.55 | 61.28 |
| >581 | 84.47 | 61.39 |
| >590 | 84.4 | 61.83 |
| >601 | 84.25 | 61.94 |
| >606 | 84.18 | 62.16 |
| >611 | 83.96 | 62.27 |
| >628 | 83.66 | 63.04 |
| >635 | 83.59 | 63.26 |
| >637 | 83.52 | 63.48 |
| >648 | 83.37 | 63.7 |
| >654 | 83.3 | 63.81 |
| >667 | 83.22 | 64.36 |
| >671 | 83.15 | 64.8 |
| >676 | 83.08 | 64.91 |
| >683 | 82.78 | 65.02 |
| >687 | 82.71 | 65.24 |
| >697 | 82.63 | 65.46 |
| >704 | 82.56 | 65.68 |
| >708 | 82.49 | 65.79 |
| >713 | 82.27 | 65.9 |
| >719 | 82.19 | 66.01 |
| >721 | 82.05 | 66.12 |
| >729 | 81.97 | 66.67 |
| >735 | 81.9 | 66.78 |
| >747 | 81.82 | 66.89 |
| >758 | 81.68 | 67.33 |
| >776 | 81.6 | 67.77 |
| >784 | 81.53 | 67.99 |
| >788 | 81.46 | 68.1 |
| >796 | 81.31 | 68.21 |
| >799 | 81.24 | 68.43 |
| >802 | 81.16 | 68.54 |
| >805 | 81.09 | 68.65 |
| >808 | 81.02 | 68.76 |
| >816 | 80.94 | 69.09 |
| >819 | 80.87 | 69.2 |
| >835 | 80.72 | 69.64 |
| >838 | 80.65 | 69.86 |
| >863 | 80.5 | 70.3 |
| >867 | 80.43 | 70.41 |
| >872 | 80.35 | 70.52 |
| >897 | 80.21 | 70.85 |
| >900 | 80.13 | 71.07 |
| >902 | 80.06 | 71.29 |
| >903 | 79.91 | 71.4 |
| >914 | 79.76 | 71.51 |
| >916 | 79.69 | 71.84 |
| >924 | 79.62 | 72.28 |
| >936 | 79.47 | 72.39 |
| >959 | 79.32 | 72.94 |
| >973 | 79.25 | 73.38 |
| >984 | 79.1 | 73.6 |
| >1004 | 78.88 | 74.04 |
| >1009 | 78.73 | 74.15 |
| >1019 | 78.66 | 74.26 |
| >1023 | 78.59 | 74.37 |
| >1042 | 78.29 | 74.7 |
| >1049 | 78.22 | 74.92 |
| >1061 | 78.15 | 75.14 |
| >1082 | 78 | 75.58 |
| >1085 | 77.85 | 75.69 |
| >1121 | 77.78 | 76.35 |
| >1147 | 77.63 | 76.79 |
| >1175 | 77.34 | 76.9 |
| >1184 | 77.12 | 77.01 |
| >1218 | 76.75 | 77.12 |
| >1260 | 76.45 | 77.34 |
| >1277 | 76.38 | 77.56 |
| >1304 | 76.31 | 78.22 |
| >1322 | 76.23 | 78.44 |
| >1366 | 76.16 | 78.77 |
| >1370 | 76.09 | 78.88 |
| >1383 | 76.01 | 79.21 |
| >1389 | 75.94 | 79.43 |
| >1399 | 75.86 | 79.54 |
| >1401 | 75.79 | 79.65 |
| >1440 | 75.57 | 79.76 |
| >1460 | 75.42 | 79.98 |
| >1477 | 75.35 | 80.2 |
| >1483 | 75.28 | 80.31 |
| >1508 | 75.2 | 80.86 |
| >1526 | 74.98 | 80.97 |
| >1561 | 74.76 | 81.19 |
| >1609 | 74.47 | 81.52 |
| >1627 | 74.39 | 81.85 |
| >1672 | 74.25 | 82.07 |
| >1743 | 74.03 | 82.4 |
| >1768 | 73.88 | 82.62 |
| >1878 | 73.8 | 83.5 |
| >1919 | 73.73 | 83.61 |
| >1977 | 73.58 | 84.49 |
| >1992 | 73.51 | 84.6 |
| >1999 | 73.44 | 84.82 |
| >2013 | 73.22 | 84.93 |
| >2048 | 72.92 | 85.15 |
| >2090 | 72.7 | 85.26 |
| >2132 | 72.48 | 85.37 |
| >2136 | 72.41 | 85.48 |
| >2147 | 72.33 | 85.59 |
| >2190 | 72.26 | 85.7 |
| >2237 | 72.19 | 86.03 |
| >2248 | 72.04 | 86.14 |
| >2249 | 71.96 | 86.25 |
| >2301 | 71.74 | 86.36 |
| >2322 | 71.67 | 86.58 |
| >2339 | 71.6 | 86.69 |
| >2372 | 71.45 | 86.8 |
| >2377 | 71.3 | 86.91 |
| >2418 | 71.16 | 87.02 |
| >2439 | 71.01 | 87.24 |
| >2466 | 70.93 | 87.35 |
| >2608 | 70.71 | 88.34 |
| >2669 | 70.35 | 88.45 |
| >2706 | 70.05 | 88.56 |
| >2754 | 69.98 | 88.78 |
| >2875 | 69.68 | 89.11 |
| >2916 | 69.46 | 89.33 |
| >2952 | 69.39 | 89.88 |
| >2986 | 69.32 | 90.1 |
| >3010 | 69.24 | 90.21 |
| >3177 | 68.73 | 90.43 |
| >3221 | 68.65 | 90.65 |
| >3259 | 68.43 | 90.87 |
| >3390 | 68.29 | 91.31 |
| >3436 | 68.14 | 91.42 |
| >3483 | 68.06 | 91.64 |
| >3709 | 67.48 | 91.75 |
| >3802 | 67.11 | 91.86 |
| >4031 | 66.45 | 91.97 |
| >4178 | 66.23 | 92.08 |
| >4382 | 66.08 | 92.19 |
| >4416 | 65.93 | 92.41 |
| >4519 | 65.71 | 92.52 |
| >4703 | 65.34 | 92.63 |
| >4743 | 65.05 | 92.74 |
| >4813 | 64.83 | 92.85 |
| >4876 | 64.61 | 92.96 |
| >4961 | 64.53 | 93.07 |
| >5261 | 64.02 | 93.18 |
| >5290 | 63.8 | 93.29 |
| >5323 | 63.72 | 93.4 |
| >5441 | 63.5 | 93.51 |
| >5538 | 63.36 | 93.62 |
| >5640 | 63.21 | 93.73 |
| >5676 | 63.06 | 93.84 |
| >5753 | 62.84 | 93.95 |
| >5792 | 62.77 | 94.06 |
| >6182 | 61.74 | 94.17 |
| >6229 | 61.66 | 94.28 |
| >6346 | 61.37 | 94.39 |
| >6448 | 61.07 | 94.5 |
| >6591 | 60.78 | 94.61 |
| >6918 | 60.41 | 94.83 |
| >7043 | 60.12 | 94.94 |
| >7105 | 59.9 | 95.16 |
| >7377 | 59.46 | 95.27 |
| >7560 | 59.31 | 95.38 |
| >7852 | 58.87 | 95.49 |
| >7944 | 58.5 | 95.6 |
| >9000 | 56.51 | 95.71 |
| >9599 | 55.48 | 95.82 |
| >10278 | 54.53 | 95.93 |
| >10446 | 54.45 | 96.04 |
| >11699 | 52.32 | 96.15 |
| >12066 | 51.95 | 96.26 |
| >12237 | 51.88 | 96.37 |
| >12744 | 51.43 | 96.48 |
| >13794 | 50.04 | 96.59 |
| >14296 | 49.37 | 96.7 |
| >15520 | 47.76 | 96.81 |
| >18107 | 45.62 | 96.92 |
| >18820 | 44.81 | 97.03 |
| >19364 | 44.08 | 97.14 |
| >20313 | 43.19 | 97.25 |
| >20863 | 42.83 | 97.36 |
| >20972 | 42.6 | 97.47 |
| >22846 | 40.32 | 97.69 |
| >23767 | 39.07 | 97.8 |
| >24137 | 38.78 | 97.91 |
| >24611 | 38.34 | 98.02 |
| >25729 | 37.67 | 98.13 |
| >25875 | 37.6 | 98.24 |
| >26697 | 36.5 | 98.35 |
| >27291 | 35.91 | 98.46 |
| >31350 | 32.16 | 98.57 |
| >32959 | 30.54 | 98.68 |
| >33805 | 29.87 | 98.9 |
| >41492 | 23.47 | 99.01 |
| >42292 | 23.03 | 99.12 |
| >49923 | 17.73 | 99.23 |
| >57804 | 13.83 | 99.34 |
| >61945 | 12.14 | 99.45 |
| >64536 | 10.96 | 99.56 |
| >81852 | 6.77 | 99.67 |
| >87907 | 5.3 | 99.78 |
| >99999 | 0 | 100 |

| **Leucocyte count (LC)** | | |  |
| --- | --- | --- | --- |
| Criterion | Sensitivity | Specificity |  |
| ≥0 | 100 | 0 |  |
| >0 | 99.56 | 0.11 |  |
| >1 | 98.82 | 0.88 |  |
| >2 | 98.01 | 2.2 |  |
| >3 | 96.76 | 4.62 |  |
| >4 | 96.32 | 6.82 |  |
| >5 | 95.51 | 9.46 |  |
| >6 | 94.41 | 11.55 |  |
| >7 | 93.82 | 13.75 |  |
| >8 | 93.23 | 15.73 |  |
| >9 | 92.57 | 18.15 |  |
| >10 | 92.2 | 19.8 |  |
| >11 | 91.17 | 21.45 |  |
| >12 | 90.29 | 22.66 |  |
| >13 | 89.55 | 23.98 |  |
| >14 | 89.04 | 26.18 |  |
| >15 | 88.37 | 27.61 |  |
| >16 | 87.34 | 29.15 |  |
| >17 | 86.9 | 30.47 |  |
| >18 | 86.53 | 32.23 |  |
| >19 | 85.95 | 33.66 |  |
| >20 | 85.43 | 35.2 |  |
| >21 | 84.69 | 36.52 |  |
| >22 | 84.11 | 37.51 |  |
| >23 | 83.37 | 38.5 |  |
| >24 | 83 | 39.82 |  |
| >25 | 82.56 | 40.59 |  |
| >26 | 82.41 | 41.69 |  |
| >27 | 82.12 | 42.57 |  |
| >28 | 81.46 | 43.01 |  |
| >29 | 81.09 | 43.67 |  |
| >30 | 80.79 | 44.44 |  |
| >31 | 80.35 | 45.32 |  |
| >32 | 79.99 | 45.98 |  |
| >33 | 79.91 | 46.42 |  |
| >34 | 79.32 | 47.08 |  |
| >35 | 78.88 | 47.52 |  |
| >36 | 78.59 | 48.18 |  |
| >37 | 78.37 | 48.95 |  |
| >38 | 78.22 | 49.39 |  |
| >39 | 77.56 | 50.17 |  |
| >40 | 77.34 | 51.16 |  |
| >41 | 76.97 | 51.6 |  |
| >42 | 76.67 | 52.37 |  |
| >43 | 76.38 | 52.81 |  |
| >44 | 76.01 | 53.36 |  |
| >45 | 75.64 | 53.58 |  |
| >46 | 75.13 | 54.35 |  |
| >47 | 74.91 | 55.12 |  |
| >48 | 74.83 | 55.34 |  |
| >49 | 74.54 | 55.56 |  |
| >50 | 74.47 | 55.89 |  |
| >51 | 74.17 | 56.22 |  |
| >52 | 73.95 | 56.44 |  |
| >53 | 73.51 | 56.88 |  |
| >54 | 73.29 | 57.32 |  |
| >55 | 73.07 | 57.54 |  |
| >56 | 72.77 | 57.98 |  |
| >57 | 72.33 | 58.2 |  |
| >58 | 72.19 | 58.75 |  |
| >59 | 72.04 | 59.3 |  |
| >60 | 71.74 | 59.63 |  |
| >61 | 71.6 | 60.29 |  |
| >62 | 71.38 | 60.4 |  |
| >63 | 71.16 | 60.84 |  |
| >64 | 70.71 | 61.61 |  |
| >65 | 70.64 | 61.94 |  |
| >66 | 70.42 | 62.38 |  |
| >67 | 70.27 | 62.6 |  |
| >68 | 69.98 | 62.71 |  |
| >69 | 69.54 | 63.37 |  |
| >70 | 69.09 | 63.37 |  |
| >71 | 68.87 | 63.59 |  |
| >72 | 68.58 | 63.81 |  |
| >73 | 68.29 | 63.81 |  |
| >74 | 68.14 | 64.14 |  |
| >75 | 67.99 | 64.8 |  |
| >76 | 67.84 | 64.91 |  |
| >77 | 67.77 | 65.13 |  |
| >78 | 67.62 | 65.68 |  |
| >79 | 67.55 | 65.68 |  |
| >80 | 67.4 | 66.01 |  |
| >81 | 67.26 | 66.34 |  |
| >82 | 67.03 | 66.56 |  |
| >83 | 66.89 | 66.78 |  |
| >84 | 66.67 | 67.11 |  |
| >85 | 66.52 | 67.11 |  |
| >86 | 66.45 | 67.33 |  |
| >87 | 66 | 67.88 |  |
| >88 | 65.78 | 68.43 |  |
| >89 | 65.64 | 68.43 |  |
| >90 | 65.42 | 68.65 |  |
| >91 | 65.05 | 68.98 |  |
| >92 | 64.9 | 69.09 |  |
| >93 | 64.61 | 69.2 |  |
| >94 | 64.31 | 69.64 |  |
| >95 | 64.09 | 69.86 |  |
| >96 | 64.02 | 69.97 |  |
| >97 | 63.87 | 70.08 |  |
| >98 | 63.72 | 70.08 |  |
| >99 | 63.43 | 70.3 |  |
| >100 | 63.28 | 70.3 |  |
| >101 | 63.13 | 70.74 |  |
| >102 | 62.91 | 70.85 |  |
| >103 | 62.91 | 70.96 |  |
| >104 | 62.77 | 71.18 |  |
| >106 | 62.77 | 71.4 |  |
| >107 | 62.62 | 71.51 |  |
| >108 | 62.4 | 71.62 |  |
| >109 | 62.25 | 71.73 |  |
| >110 | 62.1 | 71.95 |  |
| >111 | 61.96 | 71.95 |  |
| >112 | 61.74 | 72.06 |  |
| >113 | 61.44 | 72.17 |  |
| >114 | 61.15 | 72.5 |  |
| >115 | 61.07 | 72.83 |  |
| >116 | 60.85 | 73.05 |  |
| >117 | 60.63 | 73.16 |  |
| >118 | 60.56 | 73.27 |  |
| >119 | 60.49 | 73.49 |  |
| >120 | 60.34 | 73.49 |  |
| >121 | 60.19 | 73.71 |  |
| >122 | 60.04 | 73.82 |  |
| >123 | 59.9 | 74.04 |  |
| >124 | 59.82 | 74.04 |  |
| >125 | 59.68 | 74.26 |  |
| >126 | 59.6 | 74.26 |  |
| >127 | 59.38 | 74.37 |  |
| >128 | 59.23 | 74.37 |  |
| >129 | 59.16 | 74.7 |  |
| >130 | 59.16 | 74.81 |  |
| >133 | 58.72 | 74.81 |  |
| >134 | 58.57 | 75.03 |  |
| >135 | 58.35 | 75.25 |  |
| >136 | 58.2 | 75.58 |  |
| >137 | 58.13 | 75.8 |  |
| >138 | 57.91 | 75.91 |  |
| >140 | 57.69 | 75.91 |  |
| >141 | 57.54 | 76.02 |  |
| >142 | 57.47 | 76.13 |  |
| >144 | 57.25 | 76.13 |  |
| >145 | 57.17 | 76.46 |  |
| >147 | 56.81 | 76.46 |  |
| >148 | 56.81 | 76.79 |  |
| >149 | 56.73 | 76.79 |  |
| >150 | 56.73 | 77.01 |  |
| >151 | 56.59 | 77.12 |  |
| >152 | 56.44 | 77.12 |  |
| >153 | 56.29 | 77.23 |  |
| >155 | 55.78 | 77.23 |  |
| >156 | 55.78 | 77.45 |  |
| >157 | 55.7 | 77.89 |  |
| >158 | 55.48 | 78 |  |
| >160 | 55.26 | 78 |  |
| >161 | 55.26 | 78.22 |  |
| >162 | 55.19 | 78.33 |  |
| >163 | 55.04 | 78.44 |  |
| >164 | 54.82 | 78.44 |  |
| >165 | 54.82 | 78.55 |  |
| >166 | 54.53 | 78.77 |  |
| >168 | 54.53 | 78.99 |  |
| >175 | 53.94 | 78.99 |  |
| >178 | 53.94 | 79.32 |  |
| >179 | 53.79 | 79.32 |  |
| >180 | 53.72 | 79.43 |  |
| >182 | 53.57 | 79.43 |  |
| >183 | 53.57 | 79.54 |  |
| >184 | 53.42 | 79.65 |  |
| >185 | 53.35 | 79.65 |  |
| >186 | 53.2 | 79.87 |  |
| >189 | 53.05 | 79.87 |  |
| >190 | 52.83 | 80.09 |  |
| >191 | 52.69 | 80.2 |  |
| >193 | 52.39 | 80.2 |  |
| >195 | 52.32 | 80.42 |  |
| >196 | 52.32 | 80.64 |  |
| >197 | 52.24 | 80.75 |  |
| >198 | 52.17 | 80.86 |  |
| >200 | 52.02 | 80.86 |  |
| >201 | 51.88 | 80.97 |  |
| >202 | 51.51 | 81.08 |  |
| >204 | 51.43 | 81.19 |  |
| >206 | 51.43 | 81.52 |  |
| >214 | 50.63 | 81.52 |  |
| >217 | 50.63 | 81.74 |  |
| >219 | 50.4 | 82.18 |  |
| >220 | 50.33 | 82.18 |  |
| >224 | 50.18 | 82.29 |  |
| >225 | 49.89 | 82.29 |  |
| >226 | 49.74 | 82.4 |  |
| >227 | 49.6 | 82.51 |  |
| >228 | 49.37 | 82.51 |  |
| >229 | 49.37 | 82.62 |  |
| >230 | 49.23 | 82.62 |  |
| >236 | 49.23 | 83.06 |  |
| >238 | 49.08 | 83.06 |  |
| >239 | 49.01 | 83.17 |  |
| >240 | 48.93 | 83.17 |  |
| >241 | 48.86 | 83.39 |  |
| >250 | 48.05 | 83.39 |  |
| >251 | 47.83 | 83.5 |  |
| >253 | 47.83 | 83.61 |  |
| >254 | 47.76 | 83.72 |  |
| >255 | 47.76 | 83.83 |  |
| >259 | 47.31 | 83.83 |  |
| >260 | 47.17 | 83.94 |  |
| >263 | 46.95 | 83.94 |  |
| >264 | 46.8 | 84.05 |  |
| >267 | 46.65 | 84.05 |  |
| >268 | 46.58 | 84.27 |  |
| >273 | 46.21 | 84.27 |  |
| >274 | 46.21 | 84.38 |  |
| >275 | 46.06 | 84.38 |  |
| >287 | 46.06 | 84.71 |  |
| >288 | 45.99 | 84.71 |  |
| >290 | 45.99 | 84.82 |  |
| >293 | 45.7 | 84.82 |  |
| >294 | 45.7 | 84.93 |  |
| >297 | 45.47 | 84.93 |  |
| >298 | 45.4 | 85.04 |  |
| >301 | 45.4 | 85.37 |  |
| >302 | 45.33 | 85.37 |  |
| >304 | 45.33 | 85.92 |  |
| >305 | 45.25 | 86.03 |  |
| >311 | 45.03 | 86.03 |  |
| >312 | 44.96 | 86.14 |  |
| >313 | 44.89 | 86.14 |  |
| >316 | 44.89 | 86.36 |  |
| >320 | 44.67 | 86.36 |  |
| >322 | 44.67 | 86.47 |  |
| >328 | 44.37 | 86.47 |  |
| >329 | 44.3 | 86.58 |  |
| >333 | 44.08 | 86.58 |  |
| >335 | 44 | 86.69 |  |
| >336 | 43.93 | 86.69 |  |
| >337 | 43.93 | 86.8 |  |
| >338 | 43.86 | 86.91 |  |
| >347 | 43.49 | 86.91 |  |
| >348 | 43.41 | 87.02 |  |
| >349 | 43.19 | 87.02 |  |
| >351 | 43.19 | 87.13 |  |
| >360 | 42.97 | 87.13 |  |
| >361 | 42.83 | 87.24 |  |
| >364 | 42.53 | 87.24 |  |
| >366 | 42.46 | 87.35 |  |
| >372 | 42.31 | 87.35 |  |
| >373 | 42.31 | 87.46 |  |
| >377 | 41.87 | 87.46 |  |
| >382 | 41.72 | 87.57 |  |
| >385 | 41.57 | 87.57 |  |
| >386 | 41.57 | 87.68 |  |
| >392 | 41.21 | 87.68 |  |
| >394 | 41.13 | 87.79 |  |
| >400 | 40.77 | 87.79 |  |
| >405 | 40.69 | 87.9 |  |
| >406 | 40.62 | 87.9 |  |
| >408 | 40.54 | 88.01 |  |
| >410 | 40.4 | 88.01 |  |
| >412 | 40.32 | 88.12 |  |
| >421 | 39.59 | 88.12 |  |
| >422 | 39.59 | 88.23 |  |
| >424 | 39.29 | 88.23 |  |
| >425 | 39.29 | 88.34 |  |
| >438 | 38.78 | 88.34 |  |
| >440 | 38.78 | 88.45 |  |
| >441 | 38.7 | 88.45 |  |
| >445 | 38.7 | 88.56 |  |
| >453 | 38.26 | 88.56 |  |
| >456 | 38.26 | 88.67 |  |
| >458 | 38.19 | 88.78 |  |
| >466 | 37.82 | 88.78 |  |
| >468 | 37.82 | 89 |  |
| >477 | 37.53 | 89 |  |
| >479 | 37.53 | 89.22 |  |
| >487 | 37.31 | 89.22 |  |
| >489 | 37.23 | 89.33 |  |
| >490 | 37.23 | 89.44 |  |
| >492 | 37.16 | 89.55 |  |
| >499 | 37.16 | 90.1 |  |
| >507 | 36.72 | 90.1 |  |
| >510 | 36.72 | 90.21 |  |
| >514 | 36.57 | 90.21 |  |
| >517 | 36.5 | 90.32 |  |
| >521 | 36.42 | 90.43 |  |
| >529 | 36.2 | 90.43 |  |
| >537 | 36.2 | 90.54 |  |
| >542 | 35.98 | 90.54 |  |
| >547 | 35.84 | 90.65 |  |
| >589 | 34.44 | 90.65 |  |
| >590 | 34.44 | 90.76 |  |
| >617 | 34 | 90.76 |  |
| >620 | 34 | 90.87 |  |
| >622 | 33.92 | 90.87 |  |
| >624 | 33.92 | 90.98 |  |
| >625 | 33.85 | 90.98 |  |
| >627 | 33.85 | 91.09 |  |
| >635 | 33.7 | 91.09 |  |
| >638 | 33.55 | 91.2 |  |
| >661 | 32.89 | 91.2 |  |
| >662 | 32.89 | 91.31 |  |
| >668 | 32.67 | 91.31 |  |
| >669 | 32.6 | 91.42 |  |
| >676 | 32.38 | 91.42 |  |
| >678 | 32.38 | 91.53 |  |
| >684 | 32.16 | 91.53 |  |
| >694 | 32.08 | 91.64 |  |
| >696 | 32.01 | 91.64 |  |
| >702 | 32.01 | 91.86 |  |
| >710 | 31.71 | 91.86 |  |
| >716 | 31.64 | 91.97 |  |
| >724 | 31.2 | 91.97 |  |
| >737 | 31.2 | 92.19 |  |
| >741 | 31.05 | 92.19 |  |
| >742 | 31.05 | 92.3 |  |
| >790 | 30.1 | 92.3 |  |
| >791 | 30.1 | 92.41 |  |
| >810 | 29.51 | 92.41 |  |
| >815 | 29.43 | 92.52 |  |
| >840 | 29.07 | 92.52 |  |
| >855 | 29.07 | 92.74 |  |
| >866 | 28.92 | 92.74 |  |
| >868 | 28.92 | 92.96 |  |
| >908 | 28.4 | 92.96 |  |
| >915 | 28.4 | 93.07 |  |
| >932 | 27.96 | 93.07 |  |
| >933 | 27.96 | 93.18 |  |
| >944 | 27.89 | 93.18 |  |
| >946 | 27.89 | 93.29 |  |
| >984 | 26.78 | 93.29 |  |
| >997 | 26.78 | 93.4 |  |
| >1071 | 25.61 | 93.4 |  |
| >1077 | 25.61 | 93.73 |  |
| >1129 | 25.09 | 93.73 |  |
| >1132 | 25.09 | 93.84 |  |
| >1154 | 24.58 | 93.84 |  |
| >1156 | 24.58 | 93.95 |  |
| >1191 | 24.06 | 93.95 |  |
| >1194 | 23.99 | 94.06 |  |
| >1245 | 23.03 | 94.06 |  |
| >1260 | 23.03 | 94.17 |  |
| >1263 | 22.88 | 94.17 |  |
| >1280 | 22.88 | 94.28 |  |
| >1296 | 22.81 | 94.28 |  |
| >1297 | 22.81 | 94.39 |  |
| >1353 | 22 | 94.39 |  |
| >1367 | 22 | 94.5 |  |
| >1379 | 21.93 | 94.5 |  |
| >1393 | 21.85 | 94.61 |  |
| >1583 | 19.5 | 94.61 |  |
| >1584 | 19.5 | 94.72 |  |
| >1640 | 18.98 | 94.72 |  |
| >1651 | 18.98 | 94.94 |  |
| >1653 | 18.91 | 94.94 |  |
| >1657 | 18.91 | 95.05 |  |
| >1686 | 18.47 | 95.05 |  |
| >1695 | 18.47 | 95.16 |  |
| >1696 | 18.4 | 95.16 |  |
| >1719 | 18.4 | 95.38 |  |
| >1781 | 17.88 | 95.38 |  |
| >1799 | 17.88 | 95.71 |  |
| >1892 | 17.29 | 95.71 |  |
| >1897 | 17.29 | 95.82 |  |
| >1978 | 17.07 | 95.82 |  |
| >1987 | 17.07 | 95.93 |  |
| >2048 | 16.7 | 95.93 |  |
| >2052 | 16.7 | 96.15 |  |
| >2128 | 16.26 | 96.15 |  |
| >2131 | 16.26 | 96.26 |  |
| >2193 | 15.67 | 96.26 |  |
| >2217 | 15.67 | 96.37 |  |
| >2313 | 15.16 | 96.37 |  |
| >2326 | 15.16 | 96.48 |  |
| >2394 | 14.72 | 96.48 |  |
| >2400 | 14.72 | 96.59 |  |
| >2463 | 14.35 | 96.59 |  |
| >2514 | 14.35 | 96.81 |  |
| >2540 | 14.13 | 96.81 |  |
| >2551 | 14.13 | 96.92 |  |
| >2678 | 13.32 | 96.92 |  |
| >2697 | 13.32 | 97.03 |  |
| >2765 | 12.95 | 97.03 |  |
| >2781 | 12.95 | 97.14 |  |
| >2883 | 12.73 | 97.14 |  |
| >2893 | 12.73 | 97.25 |  |
| >3082 | 12.07 | 97.25 |  |
| >3102 | 12.07 | 97.36 |  |
| >3109 | 11.99 | 97.36 |  |
| >3122 | 11.99 | 97.47 |  |
| >3143 | 11.92 | 97.47 |  |
| >3175 | 11.92 | 97.58 |  |
| >3418 | 11.48 | 97.58 |  |
| >3432 | 11.48 | 97.69 |  |
| >3622 | 11.26 | 97.69 |  |
| >3633 | 11.26 | 97.8 |  |
| >3795 | 10.67 | 97.8 |  |
| >3819 | 10.67 | 97.91 |  |
| >3906 | 10.3 | 97.91 |  |
| >3908 | 10.3 | 98.02 |  |
| >4971 | 8.02 | 98.02 |  |
| >5004 | 8.02 | 98.13 |  |
| >5590 | 7.21 | 98.13 |  |
| >5690 | 7.21 | 98.24 |  |
| >5805 | 6.84 | 98.24 |  |
| >5892 | 6.84 | 98.35 |  |
| >6418 | 6.03 | 98.35 |  |
| >6680 | 6.03 | 98.57 |  |
| >7215 | 5.74 | 98.57 |  |
| >7250 | 5.74 | 98.68 |  |
| >8501 | 4.78 | 98.68 |  |
| >8994 | 4.78 | 98.79 |  |
| >9503 | 4.42 | 98.79 |  |
| >9600 | 4.42 | 98.9 |  |
| >10551 | 3.9 | 98.9 |  |
| >10657 | 3.9 | 99.01 |  |
| >11350 | 3.53 | 99.01 |  |
| >11576 | 3.53 | 99.23 |  |
| >13788 | 2.94 | 99.23 |  |
| >14603 | 2.94 | 99.34 |  |
| >14700 | 2.87 | 99.34 |  |
| >14849 | 2.87 | 99.45 |  |
| >17493 | 2.13 | 99.45 |  |
| >17528 | 2.13 | 99.56 |  |
| >19689 | 1.62 | 99.56 |  |
| >20001 | 1.62 | 99.67 |  |
| >29267 | 0.81 | 99.67 |  |
| >30908 | 0.81 | 99.78 |  |
| >55736 | 0.15 | 99.78 |  |
| >57278 | 0.15 | 99.89 |  |
| >76069 | 0 | 99.89 |  |
| >78102 | 0 | 100 |  |

| **Squamous cell count (SQC)** | | |
| --- | --- | --- |
| Criterion | Sensitivity | Specificity |
| ≤0 | 7.73 | 98.79 |
| ≤1 | 18.84 | 96.59 |
| ≤2 | 27.45 | 94.17 |
| ≤3 | 34.07 | 91.86 |
| ≤4 | 40.18 | 89 |
| ≤5 | 45.25 | 86.91 |
| ≤6 | 49.08 | 83.94 |
| ≤7 | 53.57 | 81.63 |
| ≤8 | 56.81 | 78.88 |
| ≤9 | 59.31 | 76.02 |
| ≤10 | 61.37 | 74.37 |
| ≤11 | 63.36 | 71.95 |
| ≤12 | 64.46 | 70.08 |
| ≤13 | 66.37 | 67 |
| ≤14 | 67.7 | 65.79 |
| ≤15 | 68.95 | 64.03 |
| ≤16 | 70.86 | 61.72 |
| ≤17 | 71.96 | 59.52 |
| ≤18 | 73.14 | 57.65 |
| ≤19 | 74.25 | 55.78 |
| ≤20 | 75.13 | 54.35 |
| ≤21 | 75.86 | 52.7 |
| ≤22 | 76.82 | 51.27 |
| ≤23 | 77.41 | 50.17 |
| ≤24 | 78.44 | 49.28 |
| ≤25 | 79.32 | 47.85 |
| ≤26 | 79.76 | 46.42 |
| ≤27 | 80.43 | 45.32 |
| ≤28 | 81.09 | 44 |
| ≤29 | 81.6 | 42.57 |
| ≤30 | 82.12 | 41.47 |
| ≤31 | 82.63 | 40.48 |
| ≤32 | 83 | 39.38 |
| ≤33 | 83.3 | 38.72 |
| ≤34 | 83.59 | 37.95 |
| ≤35 | 84.4 | 36.52 |
| ≤36 | 84.92 | 36.08 |
| ≤37 | 85.14 | 35.75 |
| ≤38 | 85.43 | 35.09 |
| ≤39 | 85.8 | 33.99 |
| ≤40 | 86.39 | 33.11 |
| ≤41 | 86.61 | 32.23 |
| ≤42 | 86.9 | 31.24 |
| ≤43 | 87.2 | 31.02 |
| ≤44 | 87.49 | 30.47 |
| ≤45 | 88.08 | 30.03 |
| ≤46 | 88.82 | 29.37 |
| ≤47 | 89.18 | 28.93 |
| ≤48 | 89.33 | 27.72 |
| ≤49 | 89.55 | 27.17 |
| ≤50 | 89.99 | 26.73 |
| ≤51 | 90.36 | 26.51 |
| ≤52 | 90.65 | 26.18 |
| ≤53 | 90.73 | 25.41 |
| ≤54 | 90.95 | 24.64 |
| ≤55 | 91.39 | 24.2 |
| ≤56 | 91.76 | 23.98 |
| ≤57 | 91.76 | 23.87 |
| ≤58 | 91.83 | 23.65 |
| ≤59 | 91.91 | 23.32 |
| ≤60 | 92.05 | 22.77 |
| ≤61 | 92.13 | 22.44 |
| ≤62 | 92.27 | 22 |
| ≤63 | 92.35 | 21.89 |
| ≤64 | 92.42 | 21.23 |
| ≤65 | 92.49 | 20.79 |
| ≤66 | 92.64 | 20.46 |
| ≤67 | 92.72 | 20.13 |
| ≤68 | 92.94 | 19.69 |
| ≤71 | 92.94 | 18.59 |
| ≤72 | 93.38 | 18.15 |
| ≤73 | 93.38 | 17.38 |
| ≤74 | 93.52 | 17.16 |
| ≤75 | 93.67 | 17.16 |
| ≤76 | 93.82 | 16.72 |
| ≤77 | 93.82 | 16.39 |
| ≤78 | 94.04 | 16.06 |
| ≤79 | 94.04 | 15.62 |
| ≤80 | 94.11 | 15.4 |
| ≤81 | 94.19 | 15.29 |
| ≤82 | 94.19 | 14.85 |
| ≤83 | 94.26 | 14.52 |
| ≤85 | 94.33 | 14.41 |
| ≤86 | 94.41 | 14.08 |
| ≤87 | 94.41 | 13.64 |
| ≤88 | 94.48 | 13.42 |
| ≤89 | 94.7 | 13.31 |
| ≤90 | 94.7 | 13.2 |
| ≤91 | 94.78 | 13.09 |
| ≤92 | 94.85 | 12.87 |
| ≤95 | 95.22 | 12.65 |
| ≤97 | 95.22 | 12.21 |
| ≤99 | 95.44 | 12.21 |
| ≤102 | 95.51 | 12.1 |
| ≤103 | 95.73 | 12.1 |
| ≤105 | 95.73 | 11.66 |
| ≤108 | 96.03 | 11.66 |
| ≤109 | 96.1 | 11.55 |
| ≤110 | 96.25 | 11.55 |
| ≤111 | 96.32 | 11.22 |
| ≤112 | 96.39 | 11.22 |
| ≤115 | 96.39 | 10.45 |
| ≤116 | 96.47 | 10.23 |
| ≤117 | 96.54 | 10.12 |
| ≤119 | 96.54 | 9.68 |
| ≤120 | 96.69 | 9.68 |
| ≤121 | 96.76 | 9.57 |
| ≤133 | 96.76 | 8.36 |
| ≤134 | 96.84 | 8.36 |
| ≤143 | 96.84 | 7.26 |
| ≤144 | 96.98 | 7.04 |
| ≤146 | 96.98 | 6.82 |
| ≤148 | 97.06 | 6.82 |
| ≤149 | 97.13 | 6.71 |
| ≤150 | 97.13 | 6.6 |
| ≤151 | 97.2 | 6.38 |
| ≤153 | 97.2 | 6.27 |
| ≤154 | 97.35 | 6.16 |
| ≤160 | 97.35 | 5.39 |
| ≤163 | 97.57 | 5.39 |
| ≤165 | 97.57 | 5.17 |
| ≤167 | 97.65 | 5.17 |
| ≤169 | 97.65 | 4.95 |
| ≤172 | 97.72 | 4.95 |
| ≤175 | 97.72 | 4.73 |
| ≤176 | 97.87 | 4.73 |
| ≤177 | 97.87 | 4.62 |
| ≤180 | 97.94 | 4.51 |
| ≤182 | 98.01 | 4.29 |
| ≤189 | 98.31 | 4.29 |
| ≤211 | 98.31 | 3.3 |
| ≤214 | 98.38 | 3.08 |
| ≤218 | 98.45 | 2.97 |
| ≤219 | 98.53 | 2.86 |
| ≤230 | 98.53 | 2.64 |
| ≤232 | 98.6 | 2.64 |
| ≤237 | 98.6 | 2.42 |
| ≤241 | 98.68 | 2.42 |
| ≤245 | 98.75 | 2.2 |
| ≤255 | 98.75 | 1.76 |
| ≤275 | 98.97 | 1.76 |
| ≤287 | 98.97 | 1.43 |
| ≤290 | 99.04 | 1.43 |
| ≤311 | 99.04 | 1.21 |
| ≤317 | 99.19 | 1.1 |
| ≤320 | 99.26 | 0.99 |
| ≤368 | 99.63 | 0.99 |
| ≤373 | 99.63 | 0.77 |
| ≤378 | 99.71 | 0.77 |
| ≤379 | 99.71 | 0.55 |
| ≤395 | 99.85 | 0.55 |
| ≤428 | 99.85 | 0.22 |
| ≤562 | 99.93 | 0.22 |
| ≤1121 | 99.93 | 0 |
| ≤2946 | 100 | 0 |

| **UTI Score** | | |
| --- | --- | --- |
| Criterion | Sensitivity | Specificity |
| ≥7.82 | 100 | 0 |
| >7.82 | 100 | 0.44 |
| >8.96 | 99.78 | 0.55 |
| >9.62 | 99.78 | 0.88 |
| >10.05 | 99.63 | 0.99 |
| >10.48 | 99.56 | 1.54 |
| >11.17 | 99.34 | 1.76 |
| >11.57 | 99.27 | 1.98 |
| >12.04 | 99.27 | 2.09 |
| >12.21 | 99.2 | 2.53 |
| >12.73 | 99.12 | 3.3 |
| >12.9 | 99.05 | 3.85 |
| >13.41 | 98.98 | 4.18 |
| >13.46 | 98.9 | 6.05 |
| >13.64 | 98.9 | 6.27 |
| >13.7 | 98.76 | 6.49 |
| >13.8 | 98.61 | 7.15 |
| >14.2 | 98.46 | 7.92 |
| >14.37 | 98.46 | 8.69 |
| >14.4 | 98.24 | 10.45 |
| >14.43 | 98.24 | 10.56 |
| >14.66 | 98.17 | 11 |
| >15.26 | 98.17 | 13.31 |
| >15.28 | 98.02 | 13.97 |
| >15.36 | 98.02 | 14.52 |
| >15.62 | 97.95 | 16.5 |
| >15.66 | 97.81 | 17.27 |
| >15.75 | 97.81 | 17.38 |
| >15.8 | 97.73 | 19.03 |
| >16.01 | 97.73 | 20.9 |
| >16.25 | 97.59 | 21.34 |
| >16.33 | 97.29 | 22.66 |
| >16.36 | 97.22 | 22.66 |
| >16.81 | 97.07 | 22.88 |
| >16.87 | 96.85 | 23.98 |
| >16.98 | 96.78 | 24.2 |
| >17.01 | 96.71 | 25.08 |
| >17.09 | 96.49 | 27.61 |
| >17.51 | 96.42 | 28.49 |
| >17.53 | 96.27 | 29.92 |
| >17.55 | 96.2 | 30.69 |
| >17.57 | 96.12 | 31.9 |
| >17.67 | 96.05 | 32.12 |
| >17.76 | 95.98 | 33.99 |
| >17.82 | 95.98 | 34.1 |
| >17.88 | 95.83 | 34.65 |
| >18 | 95.68 | 35.75 |
| >18.15 | 95.61 | 35.86 |
| >18.23 | 95.54 | 36.08 |
| >18.6 | 95.54 | 36.19 |
| >18.73 | 95.17 | 39.49 |
| >18.99 | 94.88 | 41.03 |
| >19.05 | 94.81 | 41.03 |
| >19.28 | 94.66 | 41.58 |
| >19.32 | 94.66 | 41.69 |
| >19.35 | 94.59 | 41.91 |
| >19.45 | 94.37 | 43.78 |
| >19.76 | 94.37 | 44.77 |
| >19.78 | 94.22 | 45.1 |
| >19.8 | 94 | 47.41 |
| >19.81 | 93.93 | 47.74 |
| >19.83 | 93.93 | 48.73 |
| >20.3 | 93.64 | 50.39 |
| >20.46 | 93.56 | 50.83 |
| >20.55 | 93.49 | 50.94 |
| >21.1 | 93.27 | 52.26 |
| >21.35 | 93.27 | 52.48 |
| >21.42 | 93.05 | 52.81 |
| >21.61 | 93.05 | 53.47 |
| >21.88 | 92.9 | 54.46 |
| >22.21 | 92.9 | 54.68 |
| >22.38 | 92.83 | 54.79 |
| >22.41 | 92.76 | 55.01 |
| >22.45 | 92.61 | 55.12 |
| >22.46 | 92.47 | 55.45 |
| >22.69 | 92.17 | 55.78 |
| >23.36 | 92.1 | 55.78 |
| >23.57 | 91.95 | 56.66 |
| >23.76 | 91.88 | 57.32 |
| >24.19 | 91.22 | 58.97 |
| >24.24 | 91.08 | 59.3 |
| >24.26 | 91 | 59.63 |
| >24.42 | 90.93 | 60.07 |
| >24.46 | 90.93 | 61.06 |
| >24.67 | 90.78 | 61.17 |
| >24.69 | 90.64 | 61.17 |
| >24.71 | 90.56 | 61.28 |
| >24.75 | 90.49 | 61.39 |
| >24.88 | 90.49 | 61.5 |
| >25.17 | 90.42 | 61.5 |
| >25.3 | 90.34 | 61.83 |
| >25.48 | 90.27 | 62.16 |
| >25.59 | 90.2 | 62.27 |
| >25.86 | 90.2 | 62.6 |
| >26.23 | 90.12 | 63.04 |
| >26.3 | 89.9 | 63.59 |
| >26.32 | 89.9 | 63.7 |
| >26.35 | 89.83 | 63.81 |
| >26.46 | 89.76 | 64.25 |
| >26.6 | 89.76 | 64.8 |
| >26.61 | 89.47 | 66.56 |
| >26.66 | 89.1 | 67.33 |
| >27.03 | 89.03 | 67.66 |
| >27.14 | 89.03 | 67.88 |
| >27.91 | 88.95 | 68.32 |
| >28.06 | 88.88 | 68.54 |
| >28.69 | 88.81 | 69.42 |
| >28.78 | 88.73 | 69.42 |
| >28.85 | 88.59 | 70.96 |
| >28.9 | 88.22 | 71.4 |
| >29.01 | 88.22 | 71.51 |
| >29.28 | 88.15 | 72.17 |
| >29.32 | 88.15 | 72.28 |
| >29.4 | 87.93 | 73.27 |
| >29.55 | 87.86 | 73.93 |
| >29.6 | 87.86 | 74.59 |
| >29.8 | 87.78 | 74.59 |
| >30.24 | 87.71 | 74.81 |
| >30.99 | 87.42 | 74.81 |
| >31.78 | 87.34 | 75.47 |
| >31.99 | 87.05 | 75.91 |
| >32.04 | 86.91 | 76.24 |
| >33 | 86.91 | 76.79 |
| >33.2 | 86.83 | 77.01 |
| >33.68 | 86.83 | 77.12 |
| >35.07 | 86.69 | 77.34 |
| >35.5 | 86.54 | 78 |
| >35.56 | 86.39 | 78 |
| >35.8 | 86.17 | 78.22 |
| >35.86 | 86.1 | 79.21 |
| >36.56 | 86.1 | 79.54 |
| >37.26 | 86.03 | 79.65 |
| >37.38 | 86.03 | 80.42 |
| >37.56 | 85.88 | 80.42 |
| >38.48 | 85.74 | 80.53 |
| >38.53 | 85.44 | 80.86 |
| >38.56 | 85.44 | 80.97 |
| >38.82 | 85.22 | 81.3 |
| >39.91 | 85.15 | 81.52 |
| >40.77 | 85.15 | 81.85 |
| >40.82 | 85.08 | 81.96 |
| >40.99 | 85.08 | 82.29 |
| >41.51 | 85 | 82.62 |
| >41.8 | 84.93 | 82.95 |
| >41.99 | 84.93 | 83.06 |
| >42.12 | 84.71 | 83.39 |
| >42.78 | 84.71 | 83.5 |
| >43.39 | 84.56 | 83.61 |
| >44.44 | 84.56 | 83.83 |
| >48.13 | 84.35 | 83.83 |
| >49.18 | 84.27 | 84.05 |
| >49.35 | 84.27 | 84.27 |
| >49.52 | 84.2 | 84.38 |
| >50.49 | 83.98 | 84.38 |
| >50.56 | 83.91 | 84.6 |
| >50.7 | 83.76 | 84.6 |
| >52.06 | 83.69 | 84.71 |
| >52.14 | 83.61 | 85.04 |
| >53.18 | 83.61 | 85.26 |
| >54.01 | 83.17 | 85.26 |
| >54.29 | 83.17 | 85.59 |
| >54.77 | 83.03 | 85.59 |
| >54.95 | 82.74 | 85.7 |
| >55.15 | 82.74 | 85.81 |
| >55.23 | 82.66 | 85.81 |
| >55.82 | 82.66 | 86.03 |
| >56.44 | 82.44 | 86.03 |
| >56.6 | 82.44 | 86.14 |
| >57.7 | 82.3 | 86.25 |
| >57.89 | 82.22 | 86.36 |
| >58.09 | 81.64 | 86.91 |
| >58.3 | 81.42 | 87.02 |
| >58.36 | 81.42 | 87.13 |
| >59.17 | 81.35 | 87.13 |
| >59.43 | 81.13 | 87.24 |
| >59.62 | 81.05 | 87.24 |
| >59.71 | 81.05 | 87.57 |
| >60.79 | 80.54 | 88.01 |
| >60.95 | 80.4 | 88.01 |
| >61.13 | 80.32 | 88.34 |
| >61.28 | 80.32 | 88.56 |
| >61.46 | 80.03 | 88.67 |
| >61.68 | 80.03 | 88.78 |
| >61.86 | 79.74 | 89.55 |
| >61.92 | 79.66 | 89.66 |
| >62.69 | 79.3 | 89.88 |
| >62.95 | 79.01 | 90.1 |
| >63.13 | 78.64 | 90.32 |
| >64.1 | 78.49 | 90.76 |
| >64.26 | 78.27 | 90.87 |
| >65.07 | 78.13 | 90.87 |
| >66 | 78.13 | 91.09 |
| >66.65 | 77.62 | 91.09 |
| >67.78 | 77.62 | 91.31 |
| >67.95 | 77.25 | 91.31 |
| >68.1 | 77.25 | 91.42 |
| >68.32 | 76.88 | 91.97 |
| >68.36 | 76.74 | 92.19 |
| >69.26 | 76.37 | 92.3 |
| >69.32 | 76.08 | 92.52 |
| >69.84 | 75.64 | 92.52 |
| >70.26 | 75.64 | 92.63 |
| >70.51 | 75.2 | 93.07 |
| >70.6 | 75.13 | 93.07 |
| >70.76 | 74.62 | 93.29 |
| >71.12 | 74.62 | 93.4 |
| >71.59 | 74.4 | 93.62 |
| >71.93 | 74.25 | 93.62 |
| >72.2 | 74.25 | 93.73 |
| >72.61 | 74.18 | 93.95 |
| >73.02 | 73.59 | 93.95 |
| >73.3 | 73.59 | 94.06 |
| >73.6 | 73.45 | 94.06 |
| >73.95 | 73.45 | 94.28 |
| >74.18 | 73.15 | 94.39 |
| >74.56 | 73.01 | 94.39 |
| >74.68 | 73.01 | 94.5 |
| >74.82 | 72.93 | 94.5 |
| >75.33 | 72.71 | 94.61 |
| >75.69 | 72.64 | 94.61 |
| >75.84 | 72.64 | 94.72 |
| >76.24 | 72.28 | 94.72 |
| >76.63 | 72.06 | 94.83 |
| >77.35 | 71.91 | 94.83 |
| >77.58 | 71.76 | 94.94 |
| >78 | 71.76 | 95.05 |
| >78.46 | 71.62 | 95.05 |
| >78.82 | 71.62 | 95.38 |
| >81.45 | 71.18 | 95.38 |
| >82.15 | 71.1 | 95.49 |
| >82.99 | 70.67 | 95.49 |
| >83 | 70.52 | 95.6 |
| >83.19 | 69.5 | 95.93 |
| >83.46 | 68.84 | 96.04 |
| >84.03 | 67.67 | 96.26 |
| >84.9 | 67.45 | 96.26 |
| >85.47 | 66.13 | 96.48 |
| >86.55 | 65.62 | 96.48 |
| >86.62 | 65.62 | 96.59 |
| >87.03 | 65.11 | 96.7 |
| >87.93 | 63.2 | 96.7 |
| >88.2 | 62.55 | 96.92 |
| >88.44 | 62.25 | 96.92 |
| >88.52 | 61.23 | 97.03 |
| >88.78 | 61.08 | 97.03 |
| >88.85 | 59.99 | 97.25 |
| >88.98 | 59.47 | 97.25 |
| >89.22 | 59.47 | 97.36 |
| >90.07 | 59.18 | 97.36 |
| >90.22 | 58.52 | 97.47 |
| >90.79 | 58.45 | 97.47 |
| >90.97 | 58.3 | 97.58 |
| >91.11 | 57.21 | 97.69 |
| >91.3 | 56.91 | 97.69 |
| >91.48 | 55.38 | 97.8 |
| >92.56 | 55.08 | 97.8 |
| >92.68 | 53.69 | 98.02 |
| >93.18 | 52.74 | 98.02 |
| >93.2 | 52.67 | 98.13 |
| >94.4 | 51.94 | 98.13 |
| >94.56 | 51.94 | 98.24 |
| >95.05 | 50.33 | 98.24 |
| >95.35 | 50.33 | 98.35 |
| >95.74 | 49.67 | 98.35 |
| >95.92 | 47.26 | 98.68 |
| >95.96 | 46.96 | 98.68 |
| >96 | 46.45 | 98.79 |
| >96.28 | 46.31 | 98.79 |
| >96.34 | 45.28 | 98.9 |
| >96.8 | 42.28 | 98.9 |
| >96.83 | 40.82 | 99.01 |
| >96.93 | 40.67 | 99.12 |
| >96.96 | 39.43 | 99.45 |
| >98.37 | 26.48 | 99.45 |
| >98.59 | 22.31 | 99.78 |
| >98.96 | 10.46 | 99.78 |
| >99.14 | 8.05 | 99.89 |
| >99.21 | 1.46 | 100 |
| >99.36 | 0 | 100 |
